# Low‐Valent Group 14 Phosphinidenide Complexes [({SIDipp}P)_2_M] Exhibit P–M pπ–pπ Interaction (M=Ge, Sn, Pb)

**DOI:** 10.1002/chem.201905061

**Published:** 2019-12-03

**Authors:** Markus Balmer, Yannick J. Franzke, Florian Weigend, Carsten von Hänisch

**Affiliations:** ^1^ Fachbereich Chemie and Wissenschaftliches Zentrum für, Materialwissenschaften (WZMW) Philipps-Universität Marburg Hans-Meerwein-Strasse 4 35032 Marburg Germany; ^2^ Institute of Physical Chemistry Karlsruhe Institute of Technology (KIT) Kaiserstrasse 12 76131 Karlsruhe Germany; ^3^ Institute of Nanotechnology Karlsruhe Institute of Technology (KIT) Hermann-von-Helmholtz-Platz 1 76344 Eggenstein-Leopoldshafen Germany

**Keywords:** N-heterocyclic carbenes, phosphinidenes, phosphinidenides, tetrylenes

## Abstract

Herein, the synthesis of new low‐valent Group 14 phosphinidenide complexes [({SIDipp}P)_2_ m] exhibiting P–M pπ–pπ interactions (SIDipp=1,3‐bis(2,6‐diisopropylphenyl)‐imidazolidin‐2‐ylidene, M=Ge, Sn, Pb), is presented. These compounds were investigated by means of structural, spectroscopic, and quantum‐chemical methods. Furthermore, the monosubstituted compounds [(SIDippP)MX]_2_ (M=Sn, X=Cl; M=Pb, X=Br) are presented, which show dimeric structures instead of multiple bonding interaction.

## Graphical abstract

**Phosphorus‐substituted tetrylenes**: The reaction of the N‐heterocyclic carbene‐stabilized phosphinidenide [(SIDipp)PK] with [(SIMes)TX_2_] (T=Ge, Sn, Pb; X=Cl, Br) yielded solely phosphorus‐substituted tetrylenes exhibiting pπ–pπ interactions. The lead derivative is the first compound with this kind of a P−Pb multiple bond.

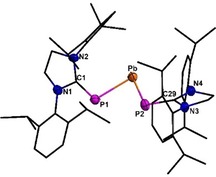

In 2010, the group of Robinson reported the synthesis of the first “parent” phosphinidene stabilized by complexation of the PH moiety with an N‐heterocyclic carbene (NHC).^1^ Since then, the synthesis of phosphinidenes and the subsequent investigation of their characteristics have been a popular research area evinced by a considerable number of publications and review articles.^2^, ^3^, ^4^, ^5^, ^6^, ^7^, ^8^, ^9^, ^10^, ^11^, ^12^, ^13^, ^14^, ^15^, ^16^, ^17^, ^18^, ^19^ Especially main‐group moieties with NHC‐ or rather cAAC‐stabilized phosphinidenide ligands (cAAC=cyclic (alkyl)(amino)carbene) are in the spotlight of recent research. Moreover, transition‐metal phosphinidenide complexes like (IDipp)PML_*n*_ (ML_*n*_=η^5^‐Cp*RuCl, η^5^‐Cp*IrCl, η^6^‐*para*‐cymene‐RuCl, and η^6^‐*para*‐cymene‐OsCl) (Cp*=C_5_Me_5_; IDipp=1,3‐bis(2,6‐diisopropylphenyl)imidazolin‐2‐ylidene) have been described by Tamm and co‐workers.^7^, ^20^ In our recent work, we focused on the synthesis of solely phosphinidenide substituted group 14 (Ge, Sn, Pb) compounds in the oxidation state +II.^2^ The group of Roesky was able to synthesize a cAAC‐stabilized silylene with two terminal phosphinidenide ligands (**I**, Scheme 1).^14^ Very recently the group of Inoue the synthesis of germylene and stannylene phosphinidene NHC complexes [^Mes^TerMP(IDipp)] (**II**) (M=Ge, Sn; ^Mes^Ter=Bis‐2,6‐(2,4,6‐trimethylphenyl)phenyl), which show a multiple M−P bond character.^21^ Only a few oligomeric compounds with bridging phosphandiide ligands like the dimeric species [{M(μ‐P{C_6_H_3_‐2,6‐(C_6_H_3_‐2,6‐*i*Pr_2_)_2_}]_2_ 22 (M=Ge, Sn, Pb), the hexameric [Ge(μ‐PSi*i*Pr_3_)]_6_ 23 or the tetrameric [Sn(μ‐PSi*t*Bu_3_)]_4_ 24 as well as other cage‐like compounds are reported.^23^, ^25^, ^26^, ^27^ As far as we know, no compound of the type M(PR)_2_ (M=Ge(+2), Sn(+2), Pb(+2)) is reported in literature. Only some rare representatives of the type M(PR_2_)_2_, like [{(Tripp)(*t*Bu)(F)Si}(*i*Pr_3_Si)P]_2_ m (M=Sn, Pb; Tripp=2,4,6‐tri‐*iso*‐propylphenyl)^28^ (**IV**) are known, which were the first crystallographically characterized diphosphanyl‐substituted tetrylenes.^28^ In contrast, the group of Izod was able to isolate [(Dipp)_2_P]_2_E and [(Tripp)_2_P]_2_E (E=Ge, Sn; Dipp=2,6‐diisopropylphenyl) (**III**), which show a significant pπ–pπ interaction apparent by a deep color of these compounds.^29^, ^30^ The compounds show one nearly planar as well as one pyramidal‐surrounded phosphorus atom, which indicates that only one pπ–pπ interaction is present. Compounds with less sterically demanding substituents tend to dimerize in solution or solid state.^31^ Furthermore, the group of Flock examined the effects that stabilize diphosphastannylenes by means of theoretical as well as experimental investigations leading to the presumption that the formation of a Sn=P double bond is less important regarding molecular stabilization. They draw the conclusion that steric shielding is the important driving force for the planarization of the phosphorus atom.^32^ In our previous work, we were able to show the possibility of the synthesis of main‐group phosphinidenide compounds through salt elimination reaction using the deprotonated compound (SIMes)PK (SIMes=1,3‐bis(2,4,6‐trimethylphenyl)imidazolidine‐2‐ylidene) as a precursor.^2^, ^3^, ^4^, ^5^

**Scheme 1:**
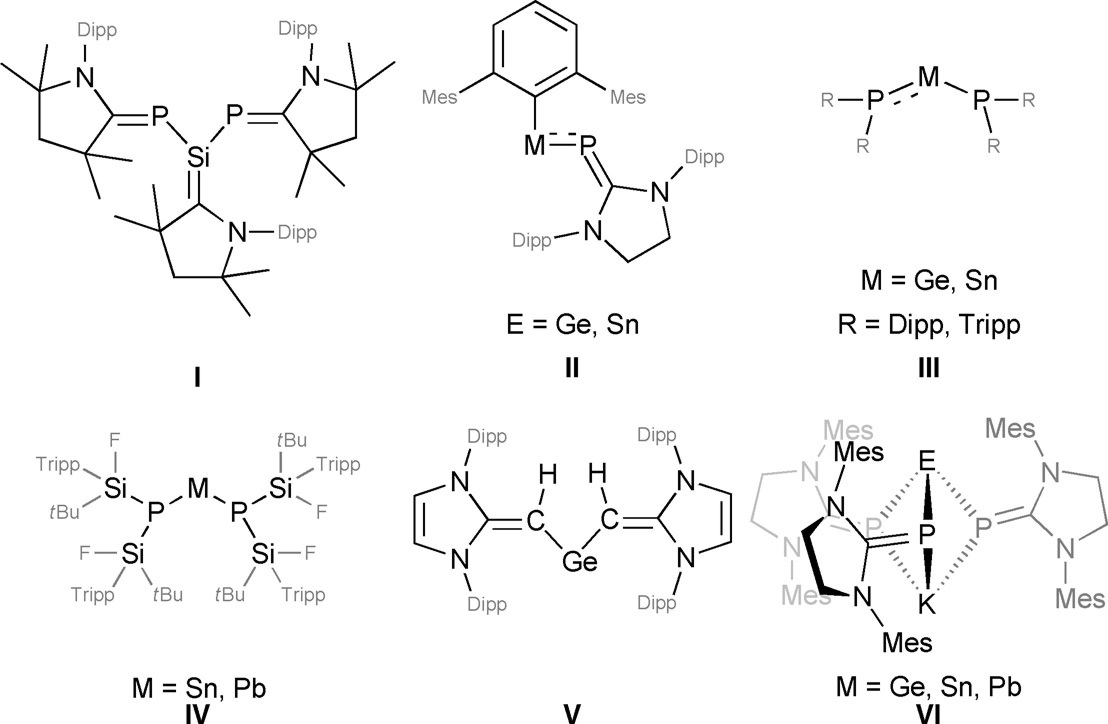
Selected examples of tetrylene compounds.

However, reactions between (SIMes)PK and (SIMes)MX_2_ (M=Ge, Sn, Pb; X=Cl or Br) yielded not the expected NHC‐stabilized phosphinidenide substituted tetrylenes. Instead, the Group 14‐ate complexes K[(SIMesP)_3_M] (M=Ge, Sn, Pb; **VI**) were formed.^2^ To avoid the formation of these ‐ate complexes, we used a new precursor with a sterically more demanding NHC ligand. The deprotonation of (SIDipp)PH (**1**) with the strong base benzyl potassium (BzK) under rigorous exclusion of air and moisture, as well as solvents containing heteroatoms (e.g. pyridine_,_ THF, and other ethers like diethyl ether), yielded (SIDipp)PK (**2**) as a red‐orange powder. As expected, the solid is completely insoluble in aliphatic or aromatic solvents (e.g. benzene, toluene, or pentane) and unstable in the presence of solvents containing heteroatoms (e.g. amines or ethers). Thus, the possibilities of characterization are rather limited. The IR spectra clearly shows that the powder is not benzyl potassium and the absence of a PH stretching mode (for (SIDipp)PH observed at $\tilde{\nu }$ =2300.3 cm^−1^) leads to the presumption that the product is the desired compound, which was confirmed with elemental analysis.

Subsequent reactions of (SIDipp)PK with (SIMes)MX_2_ (M=Ge, Sn, Pb; X=Cl or Br) at low temperatures in toluene in a 1:2 molar ratio led to deep purple colored suspensions. After removal of the formed KCl and exchange of the solvent storage of the saturated solutions in pentane at low temperatures (6 °C for **3** and **5** or −32 °C for **4**) yielded single crystals of [(SIDipp)P]_2_M (M=Ge **3**, Sn **4**, Pb **5**) as dark violet crystals in moderate yield (Scheme 2).

**Scheme 2:**
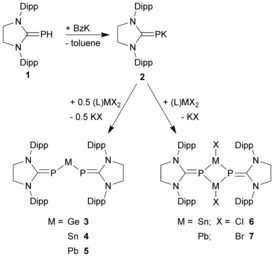
Synthesis of compounds **2**–**7**.

All three compounds crystallize isotypically in the monoclinic space group *P*2_1_/*c* with one molecule of pentane in the asymmetric unit. All compounds were characterized by ^1^H NMR, ^13^C NMR, ^31^P NMR, IR spectroscopy, and elemental analysis. It is worth mentioning that all compounds, especially compound **5**, are sensitive towards light, particularly in solution, shown by a color change from deep purple towards pale yellow, associated with precipitation of the respective metallic powder. The ^31^P NMR spectrum of **3** shows a singlet at 145.2 ppm, which is a dramatic lowfield shift compared with other germanium substituted phosphinidenides (K[(SIMesP)_3_Ge] −11.4;^2^ (IDipp)P−GePh_3_ −145.1;^18^ (SIDipp)PGePh_3_ −114.7 ppm^18^). This is in line with the trends of calculated partial charges at the M atom (see Tables S11 and S12, Supporting Information), but one should be aware, that the main effect usually comes from the differences in the response of the density to the magnetic field. This presumption is supported by the fact that this kind of lowfield shift in the ^31^P NMR spectra has also been observed for planarly coordinated phosphorus atoms with Ge=P multiple bonds (e.g. Mes_2_GePAr“, 175.4 ppm, Ar”=2,4,6‐tri‐*tert*‐butylphenyl).^33^ Another indication of a pπ–pπ interaction is the intensive color of the compounds. In contrast to the compounds [(Dipp)_2_P]_2_E and [(Tripp)_2_P]_2_E (E=Ge, Sn) of Izod and co‐workers, only one signal and no line broadening are observed in the NMR spectra indicating that the phosphorus atoms are chemically and magnetically equivalent and both are involved in the π–π interaction.^29^, ^34^ The N*C*N group shows a pseudo‐triplet splitting in the ^13^C{^1^H} NMR spectrum.

In the solid state, compound **3** (Figure 1) shows a V‐shaped structure of the GeP_2_ moiety. Comparing the P‐Ge‐P angle of **3** (87.4(1)°) with the respective ones in [(Dipp)_2_P]_2_Ge (107.40(4)°) and [(Tripp)_2_P]_2_Ge (103.98(8)°), a remarkable sharper angle is observed in **3**, which can be assigned to the greater distance between the sterically demanding substituents in **3**, because the Dipp substituents are not directly bound to the phosphorus atoms.^29^, ^30^ The Ge−P distance in **3** (229.6(1)–230.2(5) pm) is in between the short and the long one in [(Dipp)_2_P]_2_Ge (223.1(2) and 236.7(2) pm) or [(Tripp)_2_P]_2_Ge (223.37(11) and 238.23(12) pm).^29^, ^30^ This is reasonable given that germanium is probably part of two pπ–pπ interactions in **3**. However, the Ge−P distance in **3** is in good accordance with the one found in (IDipp)PGePh_3_ (228.37(4)) or [(Me_3_Si)_2_PGe(Tripp)] (229.1(4) pm).^18^, ^35^ Comparison of the C−P bond length (177.0(2)–177.3(2) pm) with other literature known compounds shows only minor differences (e.g. (IDipp)PGePh_3_ 177.48(16) pm).^18^ The average angle of the N‐C‐N plane (N1‐C1‐N2 and N3‐C28‐N4) towards the P1‐Ge‐P2 plane is 33.0°, showing a slight twist through the whole molecule. However, this slight twist does not disable a π‐interaction across the C‐P‐Ge‐P‐C moiety (see Figure S23, Supporting Information). Furthermore, this compound show many similarities with the compound [(IDippCH)_2_Ge] (**V**), which was published by Rivard and co‐workers in 2017, due to its shape as well as the analogical π‐electron density delocalized in the central C‐Ge‐C moiety.^36^ This is a vivid example for the diagonal relationship between phosphorus and carbon.

**Figure 1:**
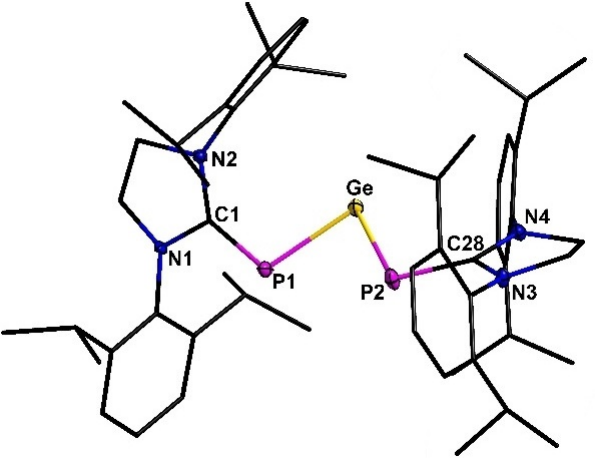
Molecular structure of **3**. Hydrogen atoms are omitted for clarity. Thermal ellipsoids for **3** represent a 50 % probability level, carbon atoms are shown as wire frame for better visibility. For selected bond lengths and angles see the Supporting Information.

Compound **4** shows a downfield shift in the ^31^P NMR spectrum (121.4 ppm with ^119^Sn satellites ^1^ *J*_119Sn−P_=1334 Hz) as observed for compound **3**. The P‐M‐P angle in **4** (85.8(1)°) is only a little bit sharper than in **3**, but again much sharper than in other literature known compounds (e.g. [(Dipp)_2_P]_2_Sn 106.20(3)°;^29^ [{(Tripp)(*t*Bu)(F)Si}(*i*Pr_3_Si)P]_2_Sn 98.78(4)°^28^) but in good accordance with [(Tripp)_2_P]_2_Sn (90.50(3)°).^29^ The Sn−P bond lengths in **4** (249.2(2)–249.9(2) pm) is between the Sn−P distance (244.58(8) pm) showing a pπ–pπ interaction in [(Dipp)_2_P]_2_Sn and the one (257.57(7) pm) which is only single bonded (Figure 2).^29^ The Sn−P distance is also significantly shorter than in other compounds without any multiple bond character (e.g. [(Tripp)_2_P]_2_Sn 256.84(9)–258.24(8) pm;^29^ [{(Tripp)(*t*Bu)(F)Si}‐(*i*Pr_3_Si)P]_2_Sn 256.7(1) pm^28^).

**Figure 2:**
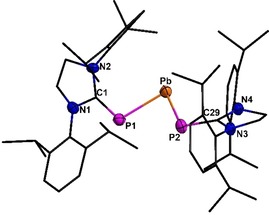
Molecular structure of **5**. Hydrogen atoms are omitted for clarity. Thermal ellipsoids for **5** represent a 50 % probability level, carbon atoms are shown as wire frame for better visibility. For selected bond lengths and angles see the Supporting Information.

Following the synthetic procedure for the germanium as well as the tin derivative, the synthesis of the lead compound (**5**) was successful. In comparison with the analytical data obtained for **3** and **4**, compound **5** meets all expectations (Table 1). The ^31^P NMR spectrum of **5** displays a signal at 116.8 ppm (^1^ *J*_Pb−P_=1673 Hz) which is once again lowfield shifted in comparison with K[(SIMesP)_3_Pb], indicating multiple‐bond character between the phosphorus and the lead atom.^2^ Again, no magnetic distinction of the phosphorus atoms is ascertainable in the ^31^P NMR spectra. As far as we know, compound **5** is the second known representative of solely twofold phosphorus‐substituted plumbylenes and the first one to exhibit pπ–pπ interaction. The other known is [{(Tripp)(*t*Bu)(F)Si}(*i*Pr_3_Si)P]_2_Pb, which was synthesized by Driess, Janoschek, and co‐workers in 1995.^37^ Since then, to the best of our knowledge no compound of this type has been published. Compound **5** exhibits notably shorter P−Pb distances (258.0(3)–258.2(3) pm) than found in [{(Tripp)(*t*Bu)(F)Si}(*i*Pr_3_Si)P]_2_Pb (265.4(4) pm), which clearly provides the assumption of a higher bond order.^37^ Other literature‐known compounds containing threefold exclusively phosphorus coordinated lead atoms show even longer P−Pb distances ([K(SIMesP)_3_Pb]: 274.5(19)–278.3(18) pm;^2^ [PbPR]_4_ with R=Si*t*Bu_2_Ph: 271.1(4)–274.6(4) pm;^38^ for R=Si(SiMe_3_): 271.5(1)–274.1(1) pm^39^ or [Pb(μ‐P*t*Bu_2_)P*t*Bu]_2_: 278.1(4)–281.2(3) pm^40^). The P‐Pb‐P angle (84.6(1)°) is slightly sharper than in **3** or **4** and but significantly sharper than in [{(Tripp)(*t*Bu)(F)Si}(*i*Pr_3_Si)P]_2_Pb (97.88(4)°),^37^ which is assigned to the steric strain exerted by the large substituents.

**Table 1:** Analytical data on compounds **3**–**5** (M=Ge, Sn, Pb).

	**3**	**4**	**5**
*d*(C−P) [pm]	177.0(2)–177.3(2)	176.5(4)–176.8(5)	174.3(10)–175.4(10)
*d*(P−M) [pm]	229.6(1)–230.2(1)	249.2(2)–249.9(2)	258.0(3)–258.2(3)
∠(C‐P‐M) [°]	104.8(1)–105.7(1)	104.1(2)–105.8(2)	103.3(4)–105.6(3)
∠(P‐M‐P) [°]	87.4(1)	85.8(3)	84.6(1)
*δ*(^31^P) [ppm}]	145.2	121.4	116.8
^1^ *J*_M−P_[Hz]	–	1334	1673
*δ*(^13^C) C_NHC_ [ppm]	191.3	192.3	186.3
First absorption maxima UV/Vis [nm]	542.5	554.0	569.0

To verify the presumption of pπ–pπ interactions between the tetrel atom and both phosphorus atoms, we performed quantum chemical calculations with the scalar‐relativistic local exact two‐component (DLU‐X2C) Hamiltonian^41^, ^42^, ^43^ employing all‐electron triple‐zeta basis sets.^44^, ^45^ Several common density functionals (see the Supporting Information) were selected together with fine grids for numerical integration^45^ and the multipole‐accelerated resolution of the identity approximation for the Coulomb term^46^ as implemented in the latest version of the TURBOMOLE program package.^47^ Based on the analytical data, the TPSSh^48^ functional performs best (see the Supporting Information for the results of all functionals). Thus, only the results with TPSSh will be discussed herein. The P−M and C−P bond lengths are overestimated by 1 and 2 pm, respectively. The trend of the ^31^P NMR shifts from Ge to Sn is in reasonable agreement with the experimental findings whereas the individual shifts are overestimated by about 20 ppm. This does not hold for compound **5** as for lead spin‐orbit effects are important for the magnetic properties.^49^, ^50^ A similar behavior is observed for the ^13^C NMR shifts. As expected, the Wiberg bond index (WBI) for the P−M bonds is greater than one for both bonds in all three compounds, especially for compound **3** (Table 2). This is a clear indicator for the multiple‐bond character. We note in passing that for the recently reported compound K[(SIMesP)_3_M]^2^ (M=Ge, Sn, Pb), in which all M−P bonds have single‐bond character, the WBI resulting from our calculations is even somewhat smaller than one (between 0.78 for M=Pb and 0.85 for M=Ge). The WBI for the P−M bonds of **3** to **5** decreases with rising atom number. In the same manner the WBI for the C−P bonds rises, indicating that these π‐bonds (C=P vs. P=M) are contrary effects. Moreover, the π‐system is delocalized over the C−P−M−P−C bonds. The effect on the C−P bond is observable in the slight shortening of d(C−P) going from **3** to **5**. Spin‐orbit effects do not significantly alter the WBI (see the Supporting Information for details on the quantum chemical calculations).

**Table 2:** Computational data (at the scalar‐relativistic x2c‐TZVPall/TPSSh functional level) of compounds **3**–**5** (M=Ge, Sn, Pb).

	**3**	**4**	**5**
WBI (C−P)	1.33	1.34	1.38
WBI (P−M)	1.20	1.13	1.08
*λ*_max abs_ [nm]	507	528	539

In all three compounds, the HOMO is represented particularly by the lone pairs at the tetrel atoms. Furthermore, there is a considerable amount of electron density at the phosphorous atoms (Figure 3). The HOMO−1 is the π‐C−P bond, the HOMO−2 (see Figure 3) is the π‐bonding combination of p orbitals of the metal atom and the phosphorus atoms, slightly deformed due to the twisting of the NHC ligands. The corresponding π*‐orbital is the LUMO orbital (see Figure 3). According to time‐dependent (TD)‐DFT^51^, ^52^, ^53^, ^54^ calculations with the DLU‐X2C Hamiltonian, the UV/Vis absorption maxima (see Table 2) correspond to singlet excitations from the HOMO−1 to the LUMO, that is, mainly from the p‐orbitals of the phosphorus atoms to the p‐orbitals of the metal center. We note that the HOMO−1 and the HOMO are close in energy (energy differences: **3**: 0.1, **4**: 0.1, **5**: 0. 05 eV) but differ in shape and symmetry. The redshift observed in the UV/Vis spectra from **3** to **5** with rising atomic number is explained by the energetic position of the p‐orbital of the metal atom, which decreases from Ge to Sn to Pb, resulting in decreasing LUMO energies from **3** to **4** to **5** and thus in decreasing excitation energies.

**Figure 3:**
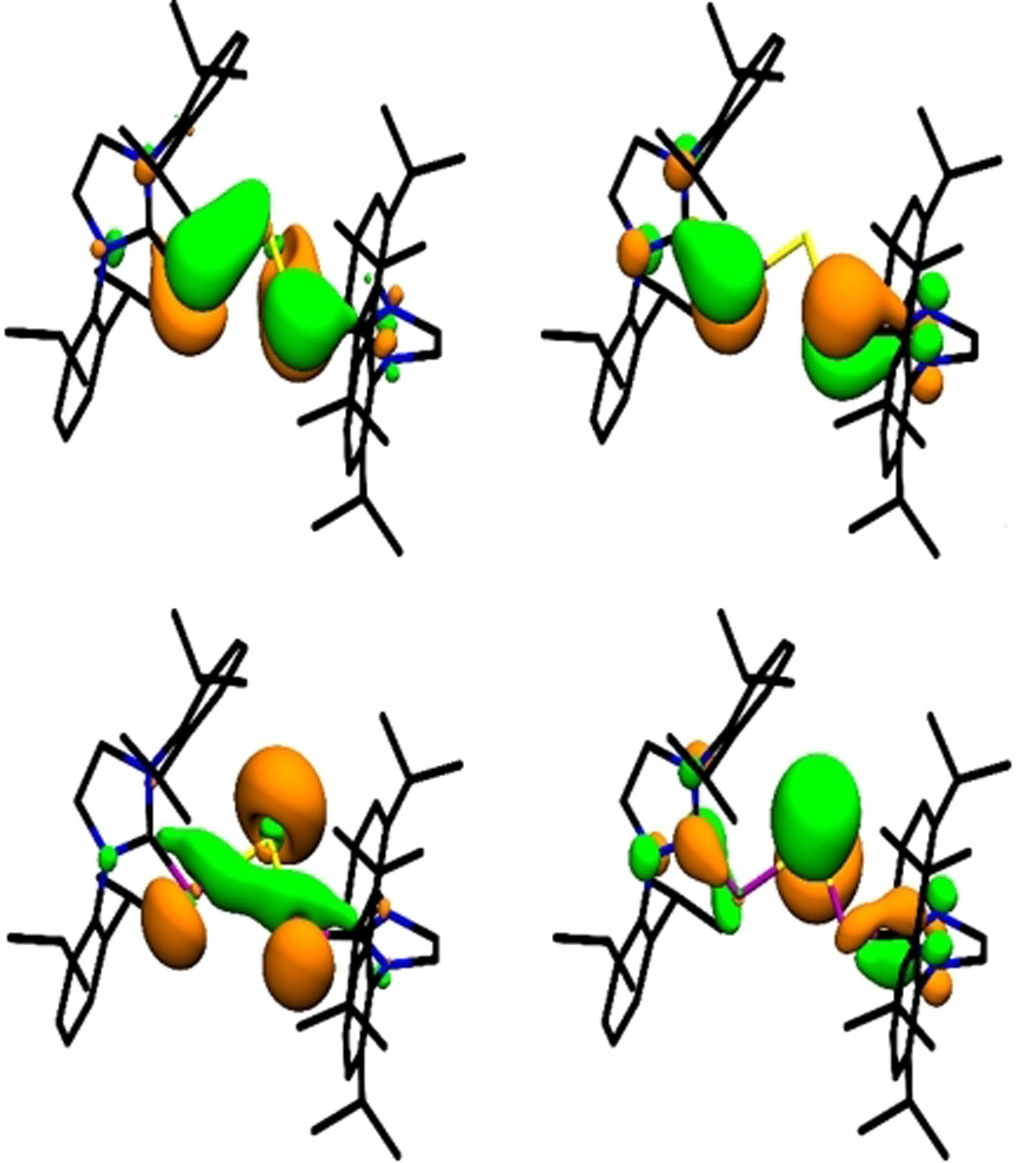
HOMO−2 (top left), HOMO−1 (top right), HOMO (bottom left) and LUMO (bottom right) of compound **4** (hydrogen atoms are omitted for clarity) with an isovalue of 0.04 a.u.

To investigate the necessity of two (SIDipp)P ligands for the formation of compounds showing pπ–pπ interaction, reactions of **2** with (SIMes)MX_2_ (M=Ge, Sn, Pb; X=Cl or Br) in a 1:1 molar ratio were performed. In all cases mixtures of (SIDippP)_2_ m, [(SIDippP)MX]_2_, (SIMes)MX_2_, SIMes, and KX were obtained. It was possible to separate (SIDippP)_2_M and SIMes (which are soluble in pentane) after changing the solvent to pentane and collection of the insoluble residue ([(SIDippP)MX]_2_, (SIMes)MX_2_ and KX). After dissolving this residue in toluene and subsequent separation of the insoluble KX through centrifugation, the isolation of [(SIDippP)SnCl]_2_ (**6**) and [(SIDippP)PbBr]_2_ (**7**) was possible in moderate yields. However, the isolation of the germanium compound was not successful because in this reaction the obtained compounds are (SIDippP)_2_Ge and (SIMes)GeCl_2_.

Compound **6** crystallizes in the monoclinic space group *P*2_1_/*n* with three molecules of toluene. In solid state, the compound forms a dimer with a central bended P_2_Sn_2_ cycle (see Figure 4). The central P_2_Sn_2_ cycle shows a butterfly conformation. Both phosphorus atoms are pyramidally coordinated by the NHC ligand and two tin atoms (sum of angles at P1: 309.8 and P2: 303.6°). The orientation of the ligands with respect to the central cycle is unusual, because two sterically demanding substituents and one chlorine ligand are situated at the same side of the ring, which leads to smaller P‐Sn2‐Cl2 angles (93.1(1) and 91.8(1)°) in comparison with the P‐Sn1‐Cl1 angles (96.0(1) and 96.6(1)°). This kind of P_2_Sn_2_ cycles are already known in the literature, but usually the tin atoms are in oxidation state Sn(+IV) (e.g. [Tripp2SnPH]^55^ or [*t*Bu2SnPH]^56^). With Sn^II^ higher aggregates like heterocubanes were formed.

**Figure 4:**
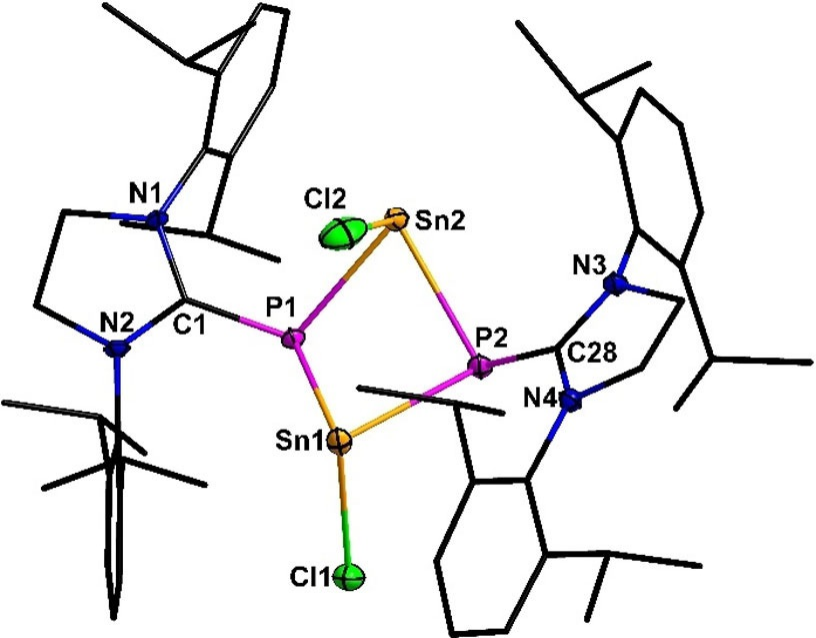
Molecular structure of **6**. Hydrogen atoms are omitted for clarity. Thermal ellipsoids for **6** represent a 50 % probability level, carbon atoms are shown as wire frame for better visibility. For selected bond lengths and angles see the Supporting Information.

The P−Sn bond lengths in **6** (259.6(1)–266.3(2) pm) are in good accordance with literature known P−Sn^II^ compounds, whereby however, P1−Sn2 is slightly shorter.^[23**]**^ Compound **6** was characterized by ^1^H NMR, ^13^C NMR, ^31^P NMR, ^119^Sn NMR, IR spectroscopy and elemental analysis (Table 3). In the ^31^P NMR spectrum, compound **6** shows a singlet signal at −66.2 ppm (^1^ *J*_Sn−P_≈1000 Hz), which is quite a usual chemical shift for tin substituted phosphinidenides.^2^ In the ^1^H NMR spectrum two signals for the isopropyl substituents are observed, which results from an inhibited rotation along the P−C bond in solution. The ^119^Sn NMR spectrum of **6** shows only one triplet signal at 235.8 ppm (^1^ *J*_P−Sn_=1027 Hz), showing that the tin atoms are equivalent on the NMR time scale and that compound **6** is a dimeric compound also in solution.

**Table 3:** Analytical data on compounds **6** and **7** (M=Sn, Pb; X=Cl, Br).

	**6**	**7**
*d*(C−P) [pm]	179.9(4)–180.1(5)	179.6(3)–179.8(3)
*d*(P−M) [pm]	259.6(1)–266.3(2)	268.9(1)–276.8(1)
*d*(M−X) [pm]	251.5(2)–254.8(2)	280.0(1)–285.0(1)
∠(C‐P‐M) [°]	103.7(2)–111.9(2)	99.4(1)–119.3(1)
∠(P‐M‐P) [°]	71.9(1)–73.3(1)	73.6(1)–75.4(1)
Σ∠M	258.2–264.5	262.0–262.2
Σ∠P	303.7–309.9	297.9–319.3
*δ*(^31^P) [ppm]	−66.2	−47.6
^1^ *J*_M−P_ [Hz]	997.8	1205.1

The reaction of **2** with (SIMes)PbBr_2_, in a 1:1 molar ratio, yielded the heavier congener [(SIDippP)PbBr]_2_ (**7**, see Figure 5). Compound **7** exhibits the same butterfly shaped central P_2_Pb_2_ cycle. Even the bromine and NHC ligands are arranged in the same manner, but the crystal structure of **7** is not isotypic, due to the lack of lattice solvent.

**Figure 5:**
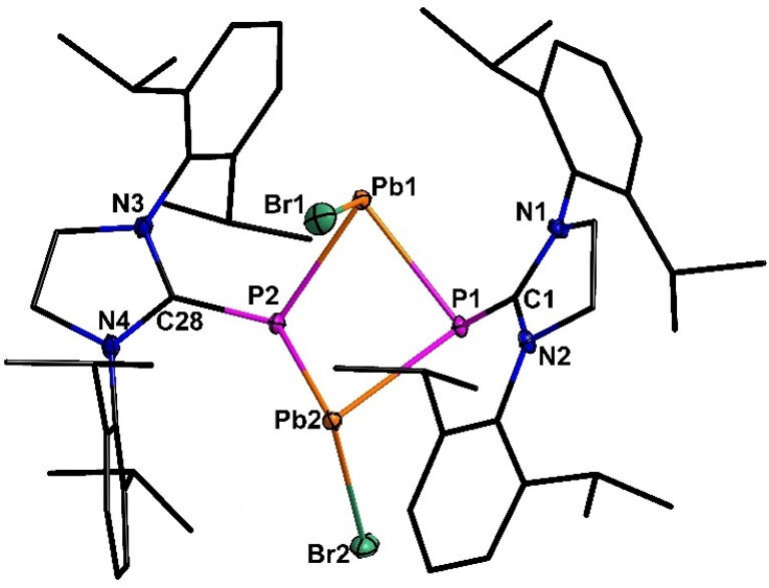
Molecular structure of **7**. Hydrogen atoms are omitted for clarity. Thermal ellipsoids for **7** represent a 50 % probability level, carbon atoms are shown as wire frame for better visibility. For selected bond lengths and angles see the Supporting Information.

The phosphorus atoms are again pyramidal surrounded by the NHC ligand and two lead atoms (sum of angles at P1: 297.9 and P2: 318.8°). The P−Pb distances (269.2(1)–276.8(1) pm) are similar to literature known compounds (e.g. [(Pb(μ‐P*t*Bu_2_)P*t*Bu_2_]_2_ 278.1(4)–281.2(3) pm;^40^ [Pb(P(SiMe_3_)_2_)_2_]_2_ 269.6(7)–279.6(7) pm;^57^ [(Me_3_Si)_3_SiPPb]_4_ 271.5(1)–274.1(1) pm^38^). Compounds that exhibit a comparable arrangement of ligands towards the central P_2_Pb_2_ cycle are the phosphanylhaloplumbylene [Fe(C_5_H_4_P*t*Bu)_2_(PbX)_2_] with X=Cl, Br, Mes) descripted by Pietschnig.^58^ The C−P bond lengths found in **7** (179.8(3)–179.6(3) pm) are in good accordance with them in **6** (180.1(5)–179.9(4) pm). In the ^31^P NMR spectrum, compound **7** displays a singlet at −47.6 ppm (^1^ *J* ${}_{{}^{207}\mathrm{Pb}-P}$ =1205 Hz), which is in the expected region of chemical shifts for phosphinidenide substituted lead(II) compounds.^[2**]**^ Compound **6** as well as **7** exhibit no pπ–pπ interaction, which can be verified by quantum chemical calculations (WBI P−M for **6**: 0.70–0.77; **7**: 0.66–0.75, for details on the calculations see the Supporting Information) and analytical data (especially ^31^P NMR spectroscopy data and P−M bond lengths).

## Conclusions

Herein we presented the new twofold phosphinidenide‐substituted tetrylenes (SIDippP)_2_M (M=Ge **3**, Sn **4**, Pb **5**) exhibiting unique pπ–pπ interaction, which resembles with the stabilization of the singlet state found in NHC ligands. As far as we know, compound **5** is the first example for this kind of interaction between phosphorous and lead atoms. For the lighter congener, only very few examples are described in literature. The character of the multiple bond between the tetrel atom and the NHC stabilized phosphinidenide was shown by means of structural, spectroscopic and quantum‐chemical methods. Furthermore, we were able to show that the twofold coordination with phosphinidenides at the tetrel is necessary, because the monosubstituted compounds [(SIDippP)MX]_2_ (M=Sn, X=Cl; M=Pb, X=Br) tend to dimerize in solution as well as in the solid state and show no sign of pπ–pπ interaction. Moreover, these compounds show the influence of the NHC ligand, since the SIDipp ligand is necessary to obtain the low valent compounds **2**–**4**. With the slightly smaller NHC substituent SIMes the *ate*‐complexes [(SIMesP)_3_M]^−^ are formed.^2^

Future investigations will focus on the reactivity of the low‐valent tetrylenes towards multiple bonds in small molecules (e.g. CO, CO_2_, or NO) as well as the coordination towards *Lewis* acids. Also, reductive cluster formation starting from the compound **6** and **7** will be a key issue. This provides access to exclusively NHC coordinated binary cage compounds.

## Conflict of interest

The authors declare no conflict of interest.

## Supporting information

As a service to our authors and readers, this journal provides supporting information supplied by the authors. Such materials are peer reviewed and may be re‐organized for online delivery, but are not copy‐edited or typeset. Technical support issues arising from supporting information (other than missing files) should be addressed to the authors.

Supplementary

Supplementary
