# Supplementary material for: Low‐Valent Group 14 Phosphinidenide Complexes [({SIDipp}P)2M] Exhibit P–M pπ–pπ Interaction (M=Ge, Sn, Pb)
Source: Chemistry. 2019 Dec 3;26(1):192–7. doi: 10.1002/chem.201905061 (PMC6972534; doi:10.1002/chem.201905061)
Supplement: Supplementary file 1 — Supplementary [file CHEM-26-192-s001.pdf]

CHEMISTRY
A European Journal

Supporting Information

Low-Valent Group 14 Phosphinidenide Complexes [({SIDipp}P)$_2$M] Exhibit P–M pπ–pπ Interaction (M = Ge, Sn, Pb)

Markus Balmer,[a] Yannick J. Franzke,[b] Florian Weigend,[c] and Carsten von Hänisch*[a]

chem_201905061_sm_miscellaneous_information.pdf

SUPPORTING INFORMATION

Table of Contents

Experimental………………………………………………………………………………………………….. S3

UV-vis spectra…………………………………………………………………………………………………. S5

NMR spectra……………………………………………………………………………………………………. S6

Compound **3** $(SIDippP)_2Ge$………………………………………………………………………….. S6

Compound **4** $(SIDippP)_2Sn$…………………………………………………………………………. S7

Compound **5** $(SIDippP)_2Pb$…………………………………………………………………………. S9

Compound **6** $[(SIDippP)SnCl]_2$……………………………………………………………….. S10

Compound **7** $[(SIDippP)PbBr]_2$………………………………………………………………… S12

X-ray structure analysis……………………………………………………………………………………. S14

Computational details……………………………………………………………………………………… S17

Analytical data at DFT level……………………………………………………………………... S17

Analysis of chemical bond: Wiberg bond indices………………………………………………… S19

Analysis of chemical bond: MO plots of frontier orbitals……………………………………….. S20

Analysis of chemical bond: Natural bond orbital population………………………………………. S25

UV-vis spectra……………………………………………………………………………………… S26

Comparison to ate complexes $K[SIMesP_3M]$……………………………………………………….. S26

References……………………………………………………………………………………………….. S27

Experimental Procedures

All manipulations were performed under an inert argon atmosphere using standard *Schlenk* techniques. The solvents were dried by standard procedures and freshly destilled before used. The storage and use of moisture or air sensitive substances were carried out under an inert argon atmosphere in a glovebox. SIDippHCl,[1] Na(PCO) · 2 dioxane,[2,3] benzyl potassium,[4] (SIMes)$GeCl_2$[5] and (SIMes)$PbBr_2$[5] were prepared according to literature methods. NMR spectra were recorded on a Bruker AV II 300 or AV III HD 300. The coupling constants *J* were reported in Hertz (Hz) and the chemical shift (δ) is given in ppm relative to the standard (^{31}P: H_3PO_4; ^{1}H, ^{13}C: $SiMe_4$; ^{119}Sn: $SnMe_4$). IR-spectra were recorded on a Bruker ALPHA FT-IR with a diamond ATR (500-4000 cm^{-1}). Elemental analyses were performed on an ELEMENTAR Vario Microcube and the content is reported in %. UV-vis spectra were recorded on an analytikjena SPECORD S600 spectrophotometer in pentane solution.

[(SIDipp)PH] (1): Compound **1** was synthesized using a modified literature known procedure.[6] A 250 mL schlenk flask was charged with SiDippHCl (24.0 g, 56.2 mmol, 1 eq.) and Na(PCO) · 2 dioxane (14.5 g, 56.2 mmol, 1 eq.) in a glovebox and 100 mL Benzonitrile was added at room temperature. The suspension was stirred at 85 °C until the reaction was completed (monitored via ^{31}P NMR spectroscopy where the signal for Na(PCO) · 2 dioxane dissappeared after approximately 35 h). The solvent of the resulting orange colored solution was completely removed under reduced pressure and slightly elevated temperatures (45 °C). The remaining solid was rigorously dried under these conditions. The solid was grinded to a fine powder in a glovebox and SIDippPH was extracted using a Soxhlett-apparatus with *n*-pentane as solvent for approximately one week. The solvent of the resulting suspension was removed and SIDippPH was obtained as a pale yellow solid. Yield 13.1 g, 30.9 mmol, 55%.

This compound is already known in literature, but was synthesized in a different manner.[7] The analytical data gathered were identical to those reported earlier.[7]

IR**:** $\tilde{\nu}$/cm^{-1} = 3066 (w), 2960 (s), 2923 (s), 2862 (m), 2300 (m, PH)**,** 1480 (s), 1453 (s), 1415 (m), 1388 (m), 1381 (m), 1351 (m), 1325 (s), 1296.2 (s), 1277 (m), 1260 (s), 1182 (s), 1092 (s), 1053 (m), 1017 (m), 937 (m), 868 (m), 799 (s), 753 (m), 703 (m), 619 (m), 558 (m), 488 (m), 428 (m).

[(SIDipp)PK] (2): The synthesis of compound **2** was inspired by literature known procedures.[8] A 250 mL schlenk flask was charged with SIDippPH (260 mg, 0.62 mmol, 1 eq.) and benzyl potassium (80 mg, 0.62 mmol, 1 eq.) in a glovebox and 100 mL toluene was added at room temperature. The reaction mixture was stirred at 40°C for 8 h. After cooling to room temperature the forming solid is gathered by filtration. The precipitation is washed three times with 20 mL toluene and 20 mL pentane. (SIDipp)PK was obtained as a red-orange powder (Yield 100 mg, 0.22 mmol, 35%). Due to the insolubility in aliphatic solvents as well as in deuterated benzene and toluene and decomposition in solvents containing heteroatoms (e.g. pyridine-d_5, THF-d_8 and other ethers like diethylether) it was not possible to analyse this compounds via NMR spectroscopy.

IR: $\tilde{\nu}$ = 2961 (m), 2934 (m), 2885 (m), 2855 (m), 1607 (w), 1472 (m), 1397 (m), 1379 (m), 1358 (m), 1292 (m), 1258 (s), 1204 (m), 1173 (m), 1094 (m), 1071 (m), 1015 (s), 930 (m), 849 (m), 801 (s), 731 (w), 696 (w), 624 (w), 597 (w), 577 (m), 527 (m), 493 (m), 466 (m) cm^{-1}.

Elemental analysis calcd. (%) for $C_{27}H_{38}N_2PK$ ([460.69 g/mol]): C 70.39, H 8.31, N 6.08; found: C 70.41, H 8.23, N 5.80.

General procedure for compounds 3-5:

(SIDipp)PK (47 mg, 0.10 mmol, 2 eq.) was added to (SIDipp)MX_2 (for M = Ge and X = Cl; M = Pb and X = Br) or TX_2 (For T = Sn and X = Cl) (0.05 mmol, 1 eq.) in 8 mL toluene at –80 °C. The reaction mixture was slowly warmed to room temperature. The solvent was removed *in vacuo* and the residue was resolved in 20 mL pentane. The suspension was centrifuged and the obtained solution was concentrated *in vacuo*. Storage of the solution at –32 °C leads to crystals, which were separated.

[(SIDipp)P_2Ge] (3): Yield 23 mg, 0.025 mmol, 49%. Suitable crystals (purple blocks) for X-ray measurements were obtained from pentane solution at 6 °C.
^{1}H NMR (300 MHz, C_6D_6): δ = 0.91 (t, $^{3}J_{H\text{-}H}$ = 7.2 Hz, 6H, C*H*$_3$ of pentane, the CH_2 groups are overlayered by CH_3 of the *i*Pr substituents), 1.25 (d, $^{3}J_{H\text{-}H}$ = 7.0 Hz, 24H, CH(C*H*$_3$)$_2$), 1.30 (d, $^{3}J_{H\text{-}H}$ = 6.8 Hz, 24H, CH(C*H*$_3$)$_2$), 3.27 (hept, $^{3}J_{H\text{-}H}$ = 6.7 Hz, 8H, C*H*(CH$_3$)$_2$), 3.53 (s, 8H, NC*H*$_2$C*H*$_2$N), 7.04 (s, 4H, C*H*$_{arom}$ para), 7.18-7.20 (m, 8H, C*H*$_{arom}$ meta) ppm.

^{13}C{^{1}H} NMR (75 MHz, C_6D_6): δ = 14.3 (s, *C*H$_3$ of pentane), 22.7 (s, *C*H$_2$CH$_3$ of pentane), 24.7 (s, CH(*C*H$_3$)$_2$), 25.3 (s, CH(*C*H$_3$)$_2$), 29.2 (s, *C*H(CH$_3$)$_2$), 34.5 (s, CH$_2$*C*H$_2$CH$_2$ of pentane) 52.6 (s, N*C*CN), 125.0 (s, *C*H$_{arom.}$ para), 129.2 (s, *C*H$_{arom.}$ meta), 137.0 (s, C$_{ipso}$), 147.6 (s, C$_{arom.}$ ortho), 191.3 (m, NCN) ppm.

^{31}P NMR (250 MHz, C_6D_6): δ = 145.2 (s) ppm.

UV-Vis (pentane solution): 542.5 , 420.0 (shoulder), 270.5, 218.5 nm.

IR: $\tilde{\nu}$/cm^{-1} = 2957 (m), 2922 (m), 2863 (m), 1587 (w), 1452 (m), 1403 (m), 1381 (m), 1346 (w), 1327 (w), 1271 (m), 1225 (s), 1194 (s), 1144 (m), 1094 (m), 1056 (m), 1023 (w), 941 (m), 797 (s), 753 (m), 727 (w), 700 (w), 615 (w), 582 (w), 551 (s), 475 (s), 417 (m).

Elemental analysis calcd. (%) for $C_{29}H_{44}P_2N_2$ ([915.81 g/mol]): C 70.82, H 8.37, N 6.12; found: C 70.31, H 8.75, N 5.96.

[[SIDipp)P$_2$Sn] (4): Yield 27 mg, 0.028 mmol, 55%. Suitable crystals (purple blocks) for X-ray measurements were obtained from toluene solution at –32 °C.

^{1}H NMR (300 MHz, C_6D_6): δ = 0.91 (t, $^3J_{HH}$ = 6.9 Hz, 6H, CH_3 of pentane, the CH_2 groups are overlayered by CH_3 of the *i*Pr substituents), 1.25 (d, $^3J_{HH}$ = 7.0 Hz, 24H, CH(CH_3)$_2$), 1.32 (d, $^3J_{HH}$ = 6.8 Hz, 24H, CH(CH_3)$_2$), 3.30 (hept, $^3J_{HH}$ = 6.8 Hz, 8H, C*H*(CH$_3$)$_2$), 3.60 (s, 8H, NCH_2CH_2N), 7.03-7.06 (m, 4H, CH_{arom} para), 7.20 (m, 8H, CH_{arom} meta) ppm.

^{13}C{^{1}H} NMR (75 MHz, C_6D_6): δ = 14.3 (s, *C*H$_3$ of pentane), 22.7 (s, *C*H$_2$CH$_3$ of pentane) , 24.7 (s, CH(*C*H$_3$)$_2$), 25.3 (s, CH(*C*H$_3$)$_2$), 29.1 (s, *C*H(CH$_3$)$_2$), 34.5 (s, CH$_2$*C*H$_2$CH$_2$ of pentane) 52.4 (s, N*CC*N), 125.1 (s, *C*H$_{arom.}$ para), 129.2 (s, *C*H$_{arom.}$ meta), 137.5 (s, C$_{ipso}$), 148.0 (s, C$_{arom.}$ Ortho), 192.3 (m, NCN) ppm.

^{31}P NMR (250 MHz, C_6D_6): δ = 121.4 (s, $^1J_{Sn\text{-}P}$ = 1334 Hz) ppm.

^{119}Sn NMR (186 MHz, C_6D_6): from +3000 ppm to -2000 ppm no signal observed.

UV-Vis (pentane solution): 554.0, 367.5, 298.5, 216.0 nm.

IR: $\tilde{\nu}$/cm^{-1} = 2958 (m), 2922 (m), 2862 (m), 1451 (m), 1396 (m), 1380 (m), 1327 (w), 1259 (m), 1226 (s), 1192 (s), 1143 (m), 1092 (s), 1055 (m), 1022 (m), 944 (m), 796 (s), 752 (m), 727 (w), 698 (w), 613 (w), 552 (s), 480 (m), 418 (w).

Elemental analysis calcd. (%) for $C_{54}H_{76}SnN_4P_2$ ([961.89 g/mol]): C 67.43, H 7.96, N 5.82; found: C 67.12, H 8.40, N 5.96.

[(SIDipp)P$_2$Pb] (5): Yield 28 mg, 0.026 mmol, 50%. Suitable crystals (purple blocks) for X-ray measurements were obtained from toluene solution at 6 °C.

^{1}H NMR (300 MHz, C_6D_6): δ = 0.91 (t, $^3J_{HH}$ = 7.1 Hz, 6H, CH_3 of pentane, the CH_2 groups are overlayered by CH_3 of the *i*Pr substituents), 1.25 (d, $^3J_{H\text{-}H}$ = 7.0 Hz, 24H, CH(CH_3)$_2$), 1.29 (d, $^3J_{H\text{-}H}$ = 6.8 Hz, 24H, CH(CH_3)$_2$), 3.28 (hept, $^3J_{H\text{-}H}$ = 6.9 Hz, 8H, C*H*(CH$_3$)$_2$), 3.73 (s, 8H, NCH_2CH_2N), 7.06 (m, 4H, CH_{arom} para), 7.20 (m, 8H, CH_{arom} meta) ppm.

^{13}C{^{1}H} NMR (75 MHz, C_6D_6): δ = 14.3 (s, *C*H$_3$ of pentane), 22.8 (s, *C*H$_2$CH$_3$ of pentane) , 24.9 (s, CH(*C*H$_3$)$_2$), 25.2 (s, CH(*C*H$_3$)$_2$), 29.0 (s, *C*H(CH$_3$)$_2$), 34.5 (s, CH$_2$*C*H$_2$CH$_2$ of pentane) 52.4 (s, N*CC*N), 125.0 (s, *C*H$_{arom.}$ para), 129.2 (s, *C*H$_{arom.}$ meta), 137.3 (s, C$_{ipso}$), 148.4 (s, C$_{arom.}$ Ortho), 186.3 (m, NCN) ppm.

^{31}P NMR (250 MHz, C_6D_6): δ = 116.8 (s, $^1J_{207Pb\text{-}P}$ = 1673 Hz) ppm.

^{207}Pb NMR (62.5 MHz, C_6D_6): from -5000 ppm to +6500 ppm no signal observed.

UV-Vis: (pentane solution): 569.0, 420.5, 244.5 (shoulder), 213.0 nm.

IR: $\tilde{\nu}$/cm^{-1} = 2957 (s), 2922 (m), 2862 (m), 1677 (w), 1586 (w), 1449 (m), 1379 (m), 1347 (w), 1327 (w), 1255 (m), 1226 (s), 1190 (s), 1143 (s), 1105 (m), 1092 (s), 1056 (m), 1024 (w), 945 (m), 797 (s), 753 (m), 727 (w), 697 (w), 614 (w), 577 (w), 553 (s), 482 (m), 428 (m).

Elemental analysis calcd. (%) for $C_{54}H_{76}PbN_4P_2$ ([1050.38 g/mol]): C 61.75, H 7.29, N 5.33; found: C 61.78, H 7.34, N 5.13.

[(SIDipp)PSnCl]$_2$ (6): (SIDipp)PK (70 mg, 0.15 mmol, 1 eq.) was added to $SnCl_2$ (29 mg, 0.15 mmol, 1 eq.) in 8 mL toluene at –80 °C. The reaction mixture was slowly warmed to room temperature. The suspension was centrifuged and the solvent of the obtained solution was removed *in vacuo*. The residue was washed twice with 20 mL pentane and afterwards resolved in toluene. Storage of the solution at –32 °C leads to crystals (yellow orange blocks), which were separated (Yield 36 mg, 0.031 mmol, 41%). Suitable crystals for X-ray measurements were obtained from a saturated solution in toluene at 6°C.

^{1}H NMR (300 MHz, C_6D_6): δ = 1.06 (d, $^3J_{HH}$ = 6.8 Hz, 24H, CH(CH_3)$_2$), 1.61 (bs, 24H, CH(CH_3)$_2$), 3.34 (hept, $^3J_{HH}$ = 6.4 Hz, 8H, C*H*(CH$_3$)$_2$), 3.40 (s, 8H, NCH_2CH_2N), 7.11-7.14 (m, 12, CH_{arom} meta+para) ppm.

^{13}C{^{1}H} NMR (75 MHz, C_6D_6): δ = 23.9 (bs, CH(*C*H$_3$)$_2$), 26.6 (s, CH(*C*H$_3$)$_2$), 29.2 (s, *C*H(CH$_3$)$_2$), 53.3 (s, N*CC*N), 130.3 (s, *C*H$_{arom.}$ para), 135.0 (s, *C*H$_{arom.}$ meta), 147.0 (s, C$_{arom.}$ ortho), not observed: C$_{Ipso}$, N*C*N ppm

^{31}P NMR (250 MHz, C_6D_6): δ = -65.3 (s, $^1J_{117Sn\text{-}P}$ = 974 Hz; $^1J_{119Sn\text{-}P}$ = 1019 Hz) ppm.

^{119}Sn NMR (186 MHz, C_6D_6): δ = 235.8 (t, $^1J_{Sn\text{-}P} \approx$ 1030 Hz) ppm.

IR: $\tilde{\nu}$/cm^{-1} = 2962 (m), 2927 (m), 2865 (w), 1630 (w), 1587 (w), 1464 (m), 1441 (m), 1418 (m), 1386 (w), 1324 (w), 1180 (w), 1147 (w), 1108 (w), 1050 (w), 948 (w), 933 (w), 798 (s), 752 (m), 730 (w), 700 (w), 623 (w), 611 (w), 552 (s), 483 (s).

Elemental analysis calcd. (%) for $C_{54}H_{76}Sn_2Cl_2N_4P_2$ ([1151.50 g/mol]): C 56.33, H 6.65, N 4.87; found: C 55.97, H 6.65, N 5.33.

[(SIDipp)PPbBr]$_2$ (7): (SIDipp)PK (70 mg, 0.15 mmol, 1 eq.) was added to (SIMes)PbBr$_2$ (102 mg, 0.15 mmol, 1 eq.) in 8 mL toluene at –80 °C. The reaction mixture was slowly warmed to room temperature. The suspension was centrifuged and the solvent of the obtained solution was removed *in vacuo*. The residue was washed twice with 20 mL pentane and afterwards resolved in toluene. Storage of the solution at –32 °C leads to crystals (yellow orange blocks), which were separated (Yield 13 mg, 0.01 mmol, 12%). Suitable crystals for X-ray measurements were obtained from a saturated solution in toluene overlayered with pentane at room temperature.

^{1}H NMR (300 MHz, C_6D_6): δ = 1.05 (d, $^3J_{HH}$ = 6.8 Hz, 24H, CH(C*H*$_3$)$_2$), 1.59k (bs, 24H, CH(C*H*$_3$)$_2$), 3.34 (hept, $^3J_{HH}$ = 6.4 Hz, 8H, C*H*(CH$_3$)$_2$), 3.467 (s, 8H, NC*H*$_2$C*H*$_2$N), 7.03-7.06 (m, 4H, C*H*$_{arom}$ para), 7.11-7.120 (m, 12, C*H*$_{arom}$ meta+para, overlayered by C_6D_6) ppm.

^{13}C{^{1}H} NMR (75 MHz, C_6D_6): δ = 23.9 (bs, CH(*C*H$_3$)$_2$), 26.6 (s, CH(*C*H$_3$)$_2$), 29.2 (s, *C*H(CH$_3$)$_2$), 53.0 (s, N*CC*N), 125.7 (bs, C$_{Ipso}$), 130.4 (s, *C*H$_{arom.}$ para), 134.7 (s, *C*H$_{arom.}$ meta), 147.5 (s, C$_{arom.}$ ortho), not observed: N*C*N ppm.

^{31}P NMR (250 MHz, C_6D_6): δ = -47.6 (s, $^1J_{207Pb\text{-}P}$ = 1205 Hz) ppm.

IR: $\tilde{\nu}$/cm^{-1} = 2958 (m), 2926 (s), 2866 (s), 1562 (m), 1466 (m), 1453 (m), 1440 (m), 1407 (m), 1386 (m), 1363 (w), 1342 (w), 1326 (w), 1267 (s), 1254 (s), 1235 (s), 1180 (w), 1146 (w), 1048 (w), 932 (w), 798 (s), 753 (m), 729 (w), 696 (w), 620 (m), 612 (m), 576 (w), 548 (s), 476 (s), 429 (w).

Elemental analysis calcd. (%) for $C_{54}H_{76}Pb_2Br_2N_4P_2$ ([1417.39 g/mol]): C 45.76, H 5.40, N 3.95; found: C 45.47, H 5.37, N 4.34.

UV/Vis spectra

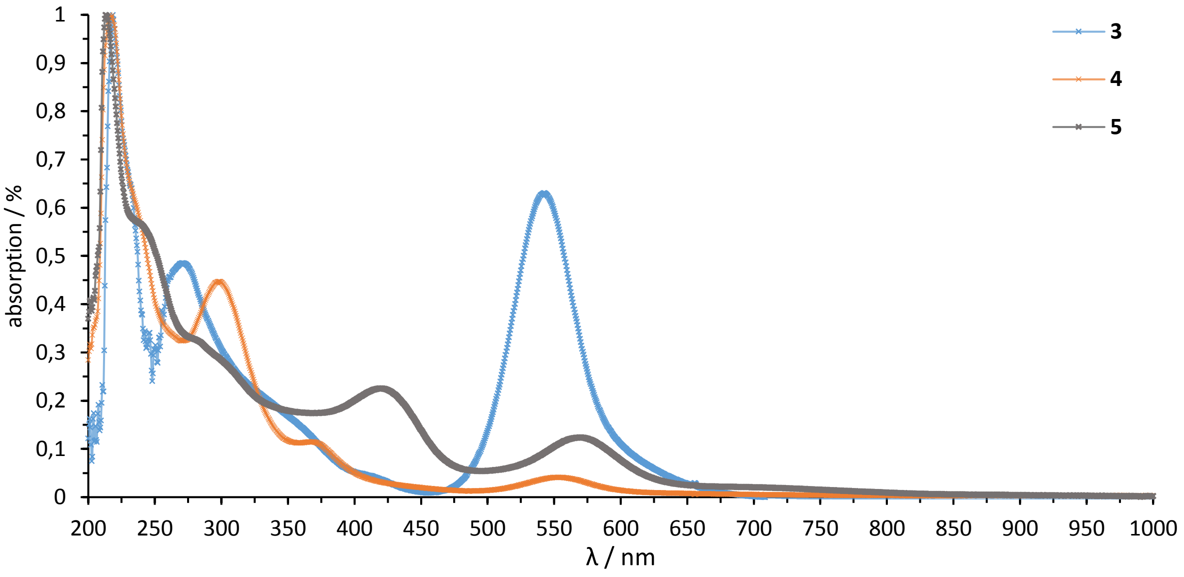

Figure S1: UV-Vis spectra of compound **3**-**5** recorded in very diluted pentane solution (the respective absorption maximum is set as one for calibration in each case).

NMR spectra

Compound 3

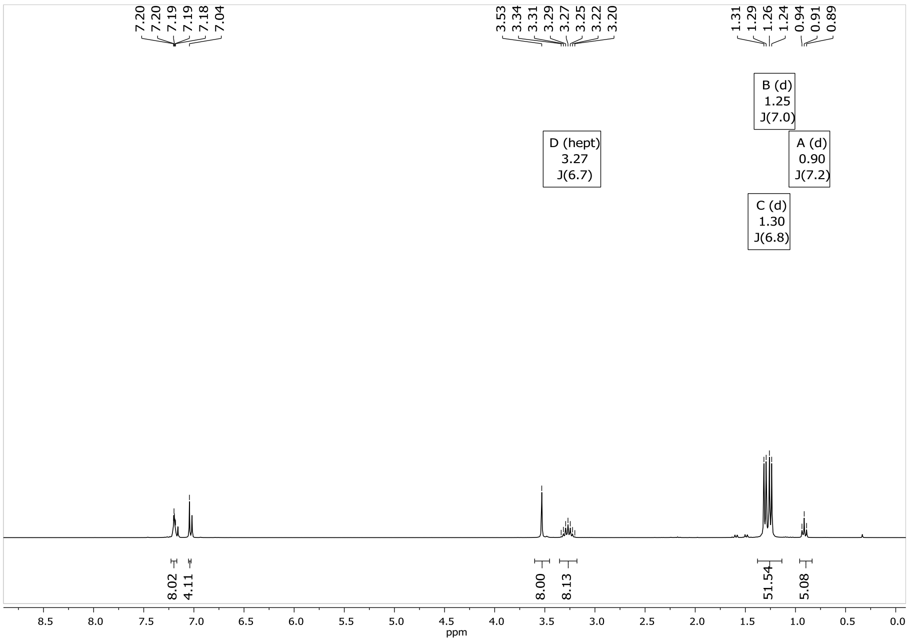

Figure S2: ^{1}H NMR spectrum of compound **3** in C_6D_6.

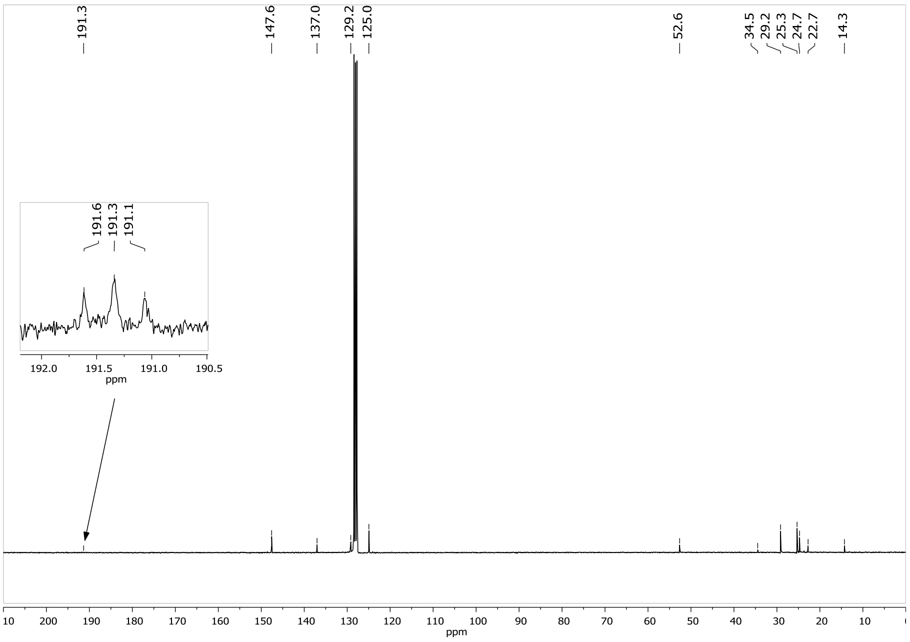

Figure S3: $^{13}C\{^{1}H\}$ NMR spectrum of compound **3** in C_6D_6.

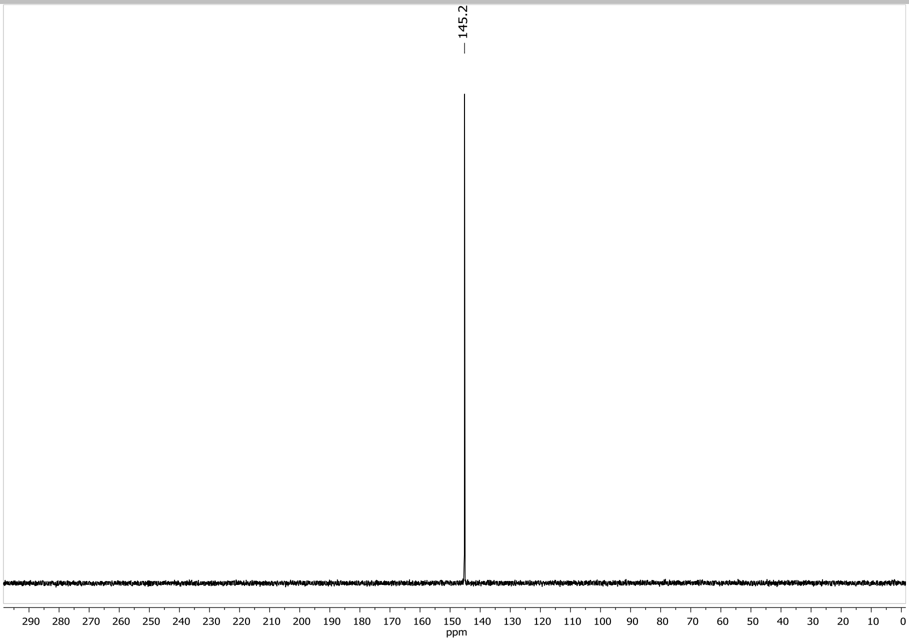

Figure S4: ^{31}P NMR spectrum of compound **3** in C_6D_6.

Compound 4

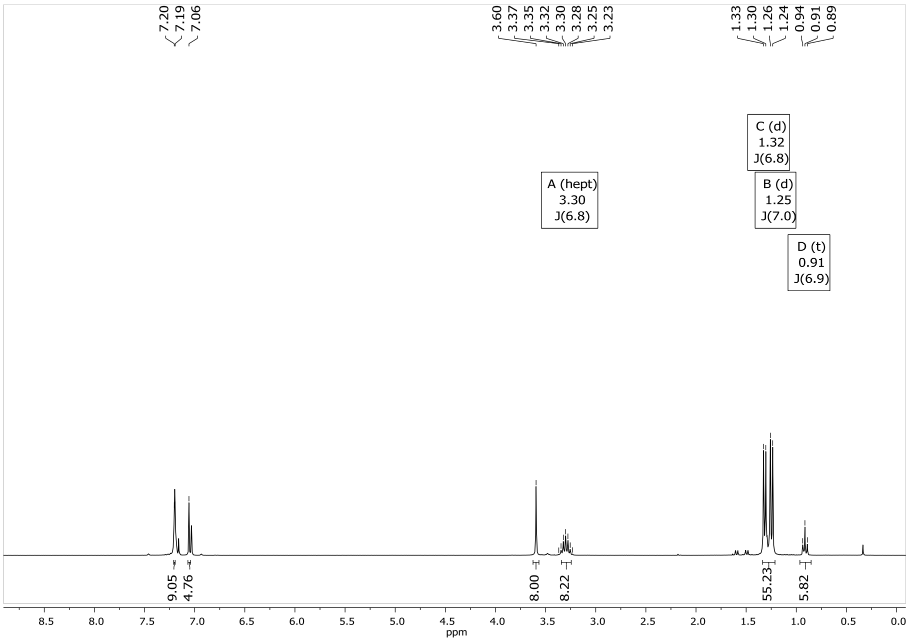

Figure S5: ^{1}H NMR spectrum of compound **4** in C_6D_6.

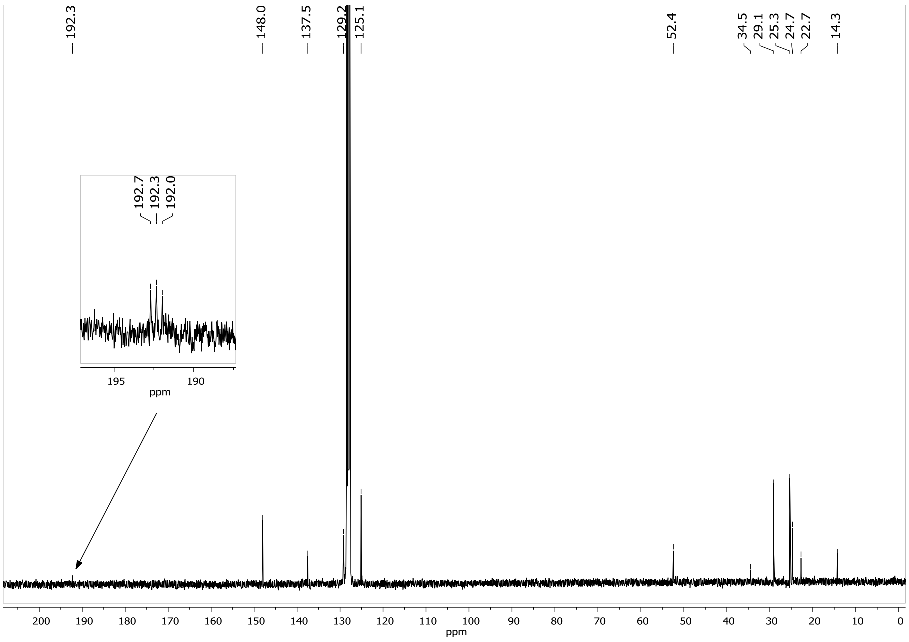

Figure S6: $^{13}C\{^{1}H\}$ NMR spectrum of compound **4** in C_6D_6.

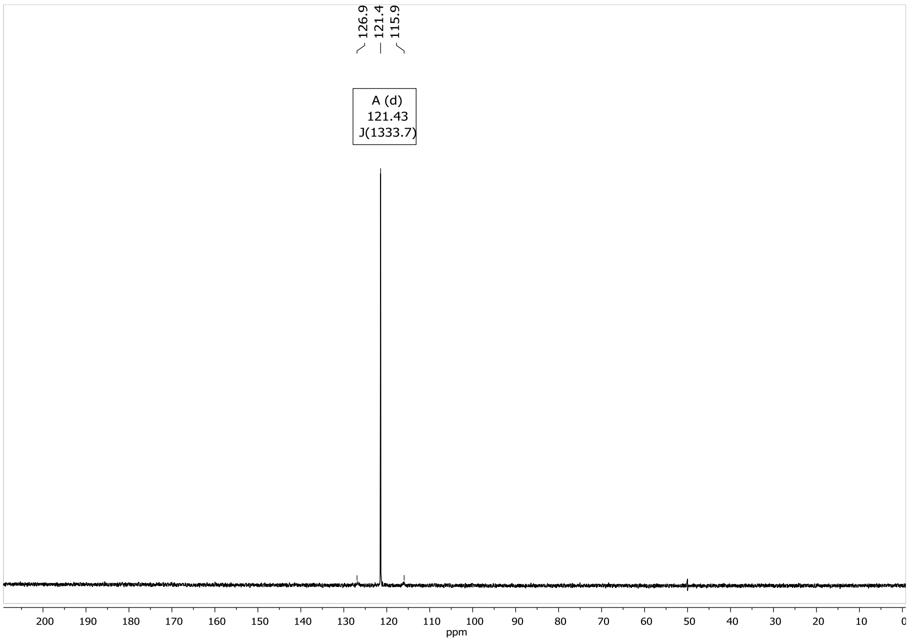

Figure S7: ^{31}P NMR spectrum of compound **4** in C_6D_6.

Compound 5

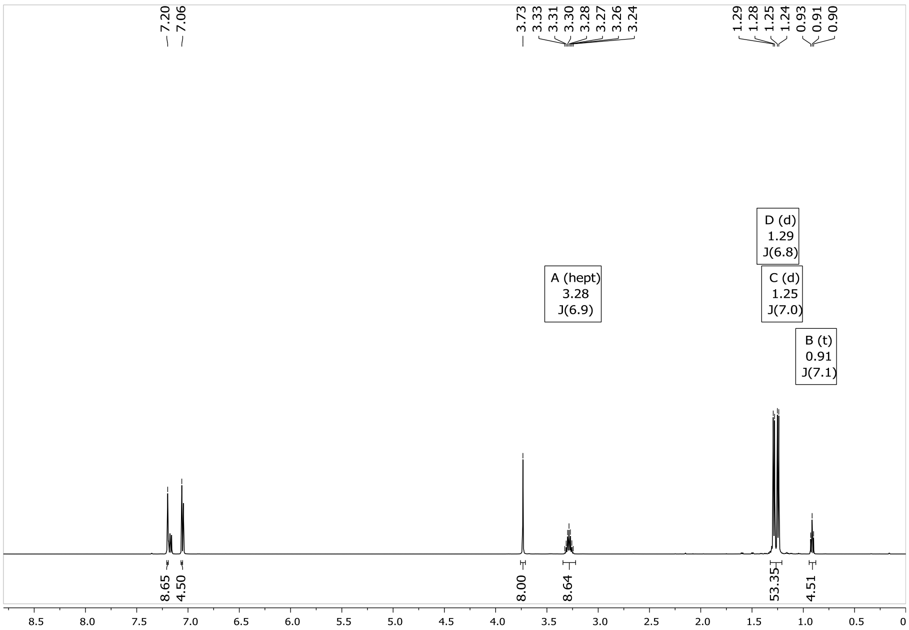

Figure S8: ^{1}H NMR spectrum of compound **5** in C_6D_6.

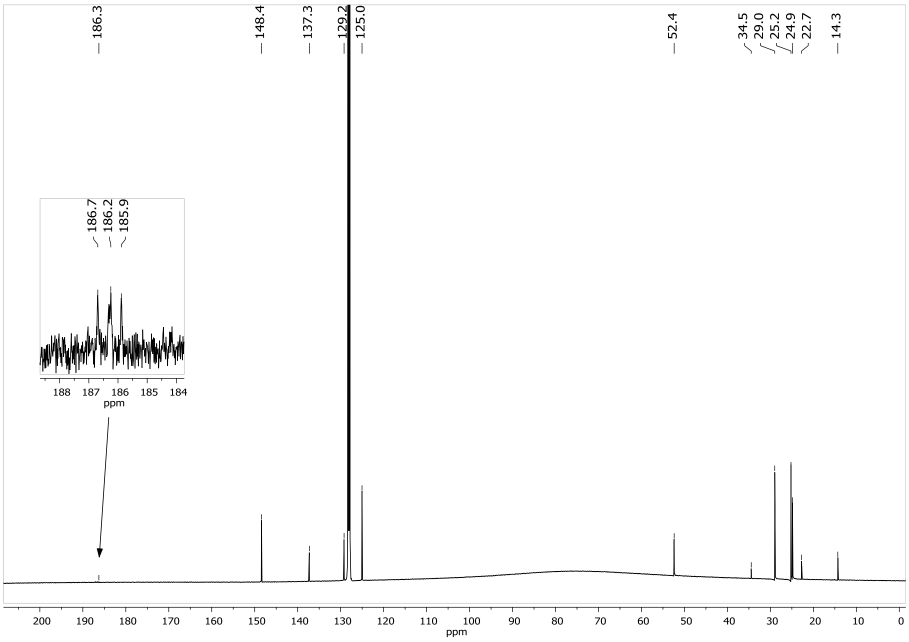

Figure S9: $^{13}C\{^{1}H\}$ NMR spectrum of compound **5** in C_6D_6.

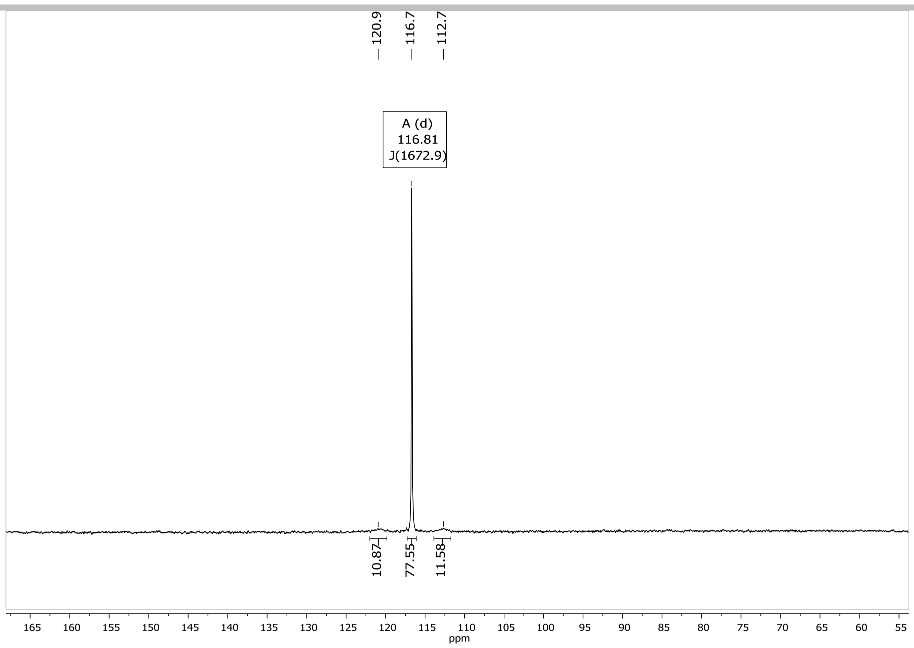

Figure S10: ^{31}P NMR spectrum of compound **5** in C_6D_6.

Compound 6

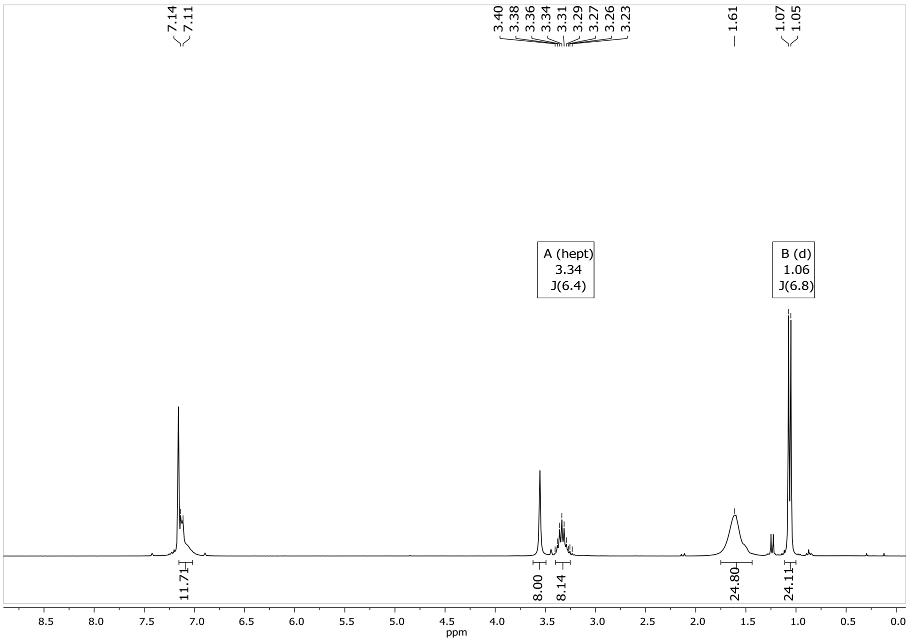

Figure S11: ^{1}H NMR spectrum of compound **6** in C_6D_6.

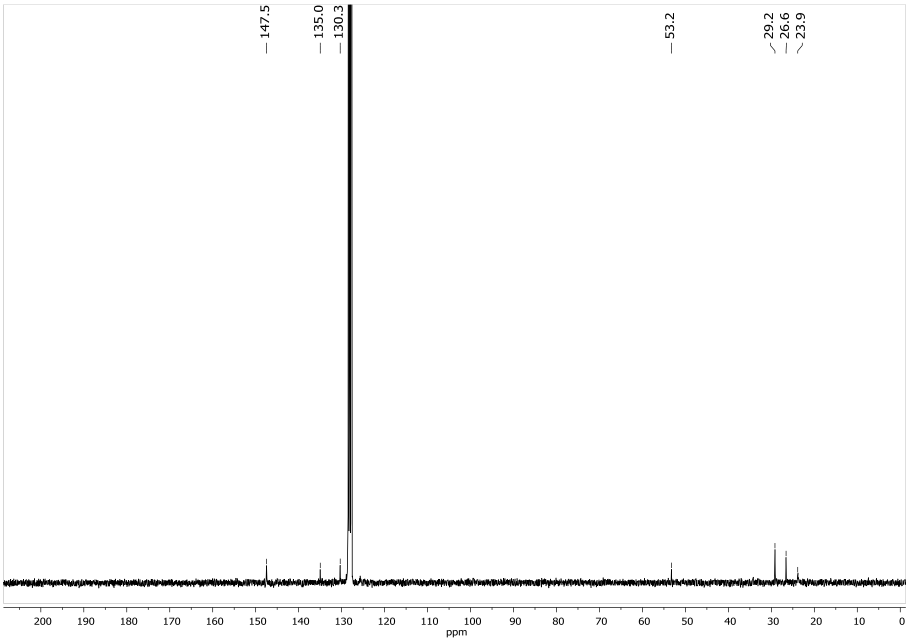

Figure S12: $^{13}C\{^{1}H\}$ NMR spectrum of compound **6** in C_6D_6.

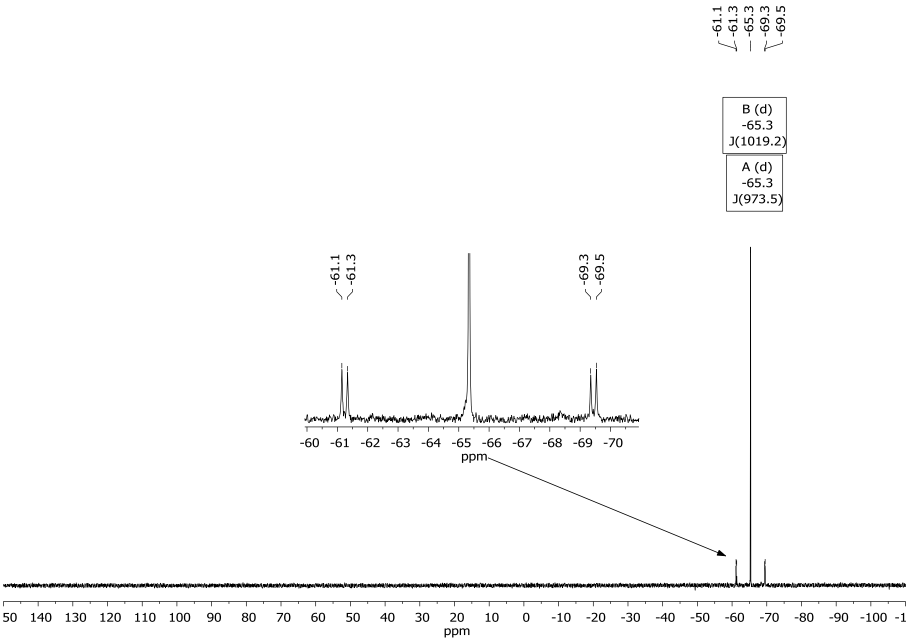

Figure S13: ^{31}P NMR spectrum of compound **6** in C_6D_6.

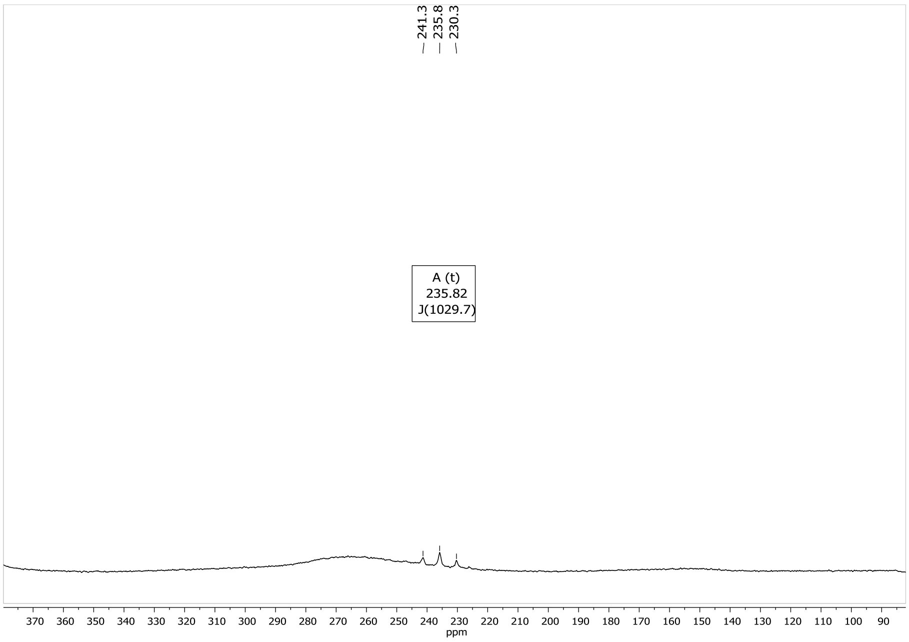

Figure S14: ^{119}Sn NMR spectrum of compound **6** in C_6D_6.

Compound 7

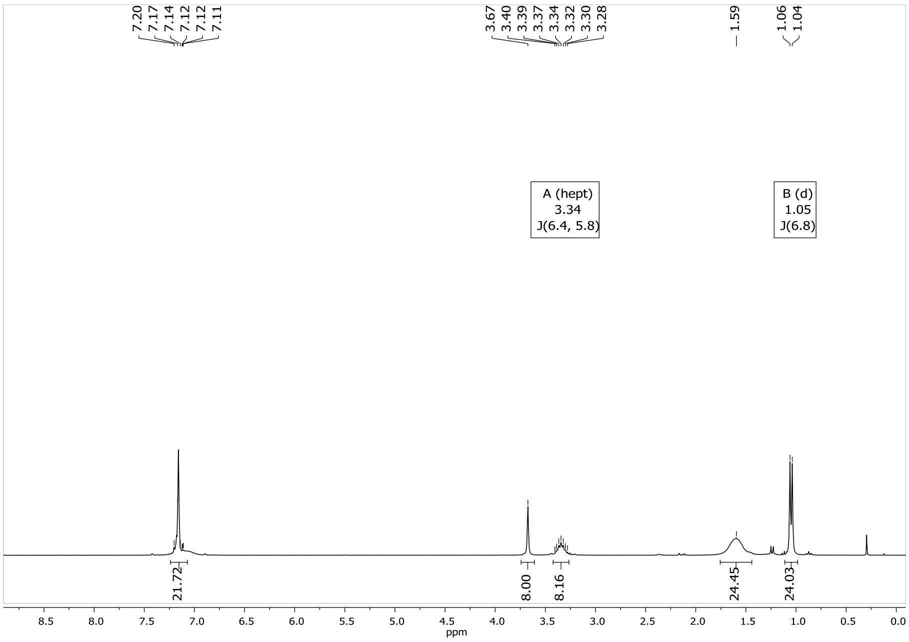

Figure S15: ^{1}H NMR spectrum of compound **7** in C_6D_6.

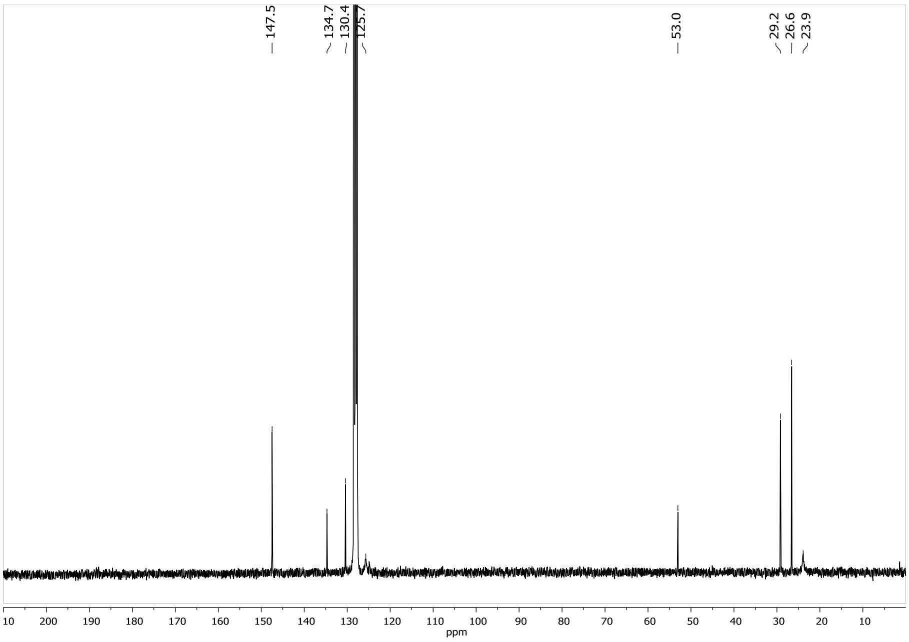

Figure S16: $^{13}C\{^{1}H\}$ NMR spectrum of compound **7** in C_6D_6.

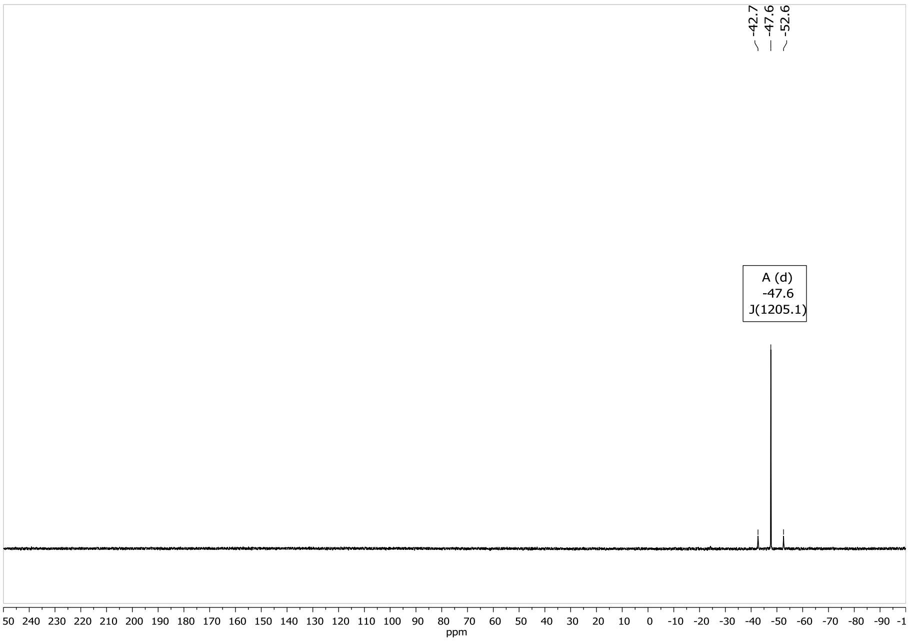

Figure S17: ^{31}P NMR spectrum of compound **7** in C_6D_6.

X-ray structure analysis

Data were collected on a Bruker D8 Quest diffractometer (compound **3**, **4**, **6** and **7**) using monochromatic Mo-Kα radiation (λ = 0.71073 Å) or on a STOE StadiVari (compound **5**) using monochromatic Cu-Kα radiation (λ = 1.54186 Å). The solution of the structure was performed with direct methods with the SHELXT-2015 solution programme, while for the structure refinement with full-matrix least-squares against F^2 the SHELXL-2015 package was used, both within the OLEX² environment.[9–11]

Crystal data of 3: $C_{54}H_{76}GeN_4P_2{\cdot}C_5H_{12}$, 987.86 g·mol^{-1}, 100 K, monoclinic, $P2_1/c$, a = 2396.9(1) pm, b = 1239.63(5) pm, c = 1967.17(8) pm, α = 90°, β = 99.801(1)°, γ = 90°, V = 5759.7(4) Å^3, Z = 4, ρ = 1.139 g·cm^{-3}, μ = 0.626, $F(000)$ = 2128.0, $GooF$ = 1.045. A total of 133466 reflections was collected of which 13254 were unique (R(int) = 0.0902). R_1 (wR_2 all data) = 0.0441 (0.0899) for 607 parameters and 10148 reflections ($I > 2\sigma(I)$). CCDC 1919176.

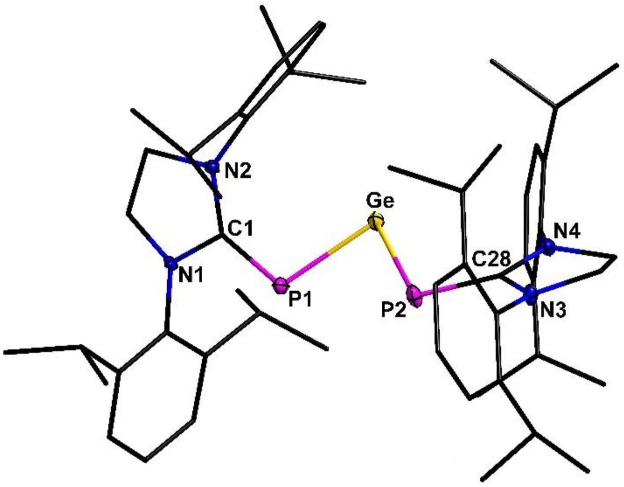

Figure S18. Molecular structure of **3**. Carbon-bound hydrogen atoms are omitted for clarity. Thermal ellipsoids for **3** represent a 50% probability level, carbon atoms are shown as sticks/wires for better visibility. Selected bond lengths /pm and angles /°: P1-Ge 230.20(5); P2-Ge 229.58(5); P1-C1 176.98(19); P2-C28 177.31(19); Ge-P1-C1 104.83(6); Ge-P2-C28 105.69(6); P1-Ge-P2 87.404(19); angle between the N1-C1-N2 and N3-C28-N4 plane 66.060(142); angle between the N1-C1-N2 and P1-Ge-P2 plane 35.837(103); angle between the N3-C28-N4 and P1-Ge-P2 plane 30.237(117).

Crystal data of 4: $C_{54}H_{76}N_4P_2Sn{\cdot}C_5H_{12}$, 1033.98 g·mol^{-1}, 100K, monoclinic, $P2_1/c$, a = 2425.05(19) pm, b = 1236.48(11) pm, c = 1966.16(16) pm, α = 90°, β = 98.785(2)°, γ = 90°, V = 5826.4(8) Å^3, Z = 4, ρ = 1.179 g·cm^{-3}, μ = 0.531, $F(000)$ = 2200.0, $GooF$ = 1.028. A total of 39479 reflections was collected of which 10386 were unique (R(int) = 0.0878). R_1 (wR_2 all data) = 0.0501 (0.0922) for 610 parameters and 7109 reflections ($I > 2\sigma(I)$). CCDC 1919174.

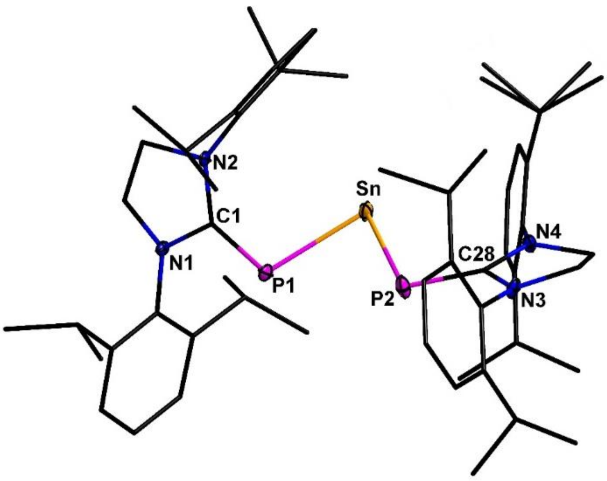

Figure S19. Molecular structure of **4**. Carbon-bound hydrogen atoms are omitted for clarity. Thermal ellipsoids for **2** represent a 50% probability level, carbon atoms are shown as sticks/wires for better visibility. Selected bond lengths /pm and angles /°: P1-Sn 249.85(11); P2-Sn 249.17(12); P1-C1 176.47(34); P2-C28 176.78(53); Sn-P1-C1 104.05(12); Sn-P2-C28 105.81(12); P1-Sn-P2 85.82(3); angle between the N1-C1-N2 and N3-C28-N4 plane 65.203(281); angle between the N1-C1-N2 and P1-Sn-P2 plane 28.578(168); angle between the N3-C28-N4 and P1-Sn-P2 plane 26.834(223).

Crystal data of 5: $C_{54}H_{76}N_4P_2Pb{\cdot}C_5H_{12}$, 1122.46 g·mol^{-1}, 100K, monoclinic, $P2_1/c$, a = 2435.4(4) pm, b = 1237.09(15) pm, c = 1961.6(3) pm, α = 90°, β = 98.676(14)°, γ = 90°, V = 5842.3(15) Å^3, Z = 4, ρ = 1.276 g·cm^{-3}, μ = 6.409, *F(000)* = 2328.0, *GooF* = 0.847. A total of 152903 reflections was collected of which 152903 were unique (R(int) = 0.2260). R_1 (wR_2 all data) = 0.0708 (0.1843) for 615 parameters and 83845 reflections ($I > 2\sigma(I)$). CCDC 1919178.

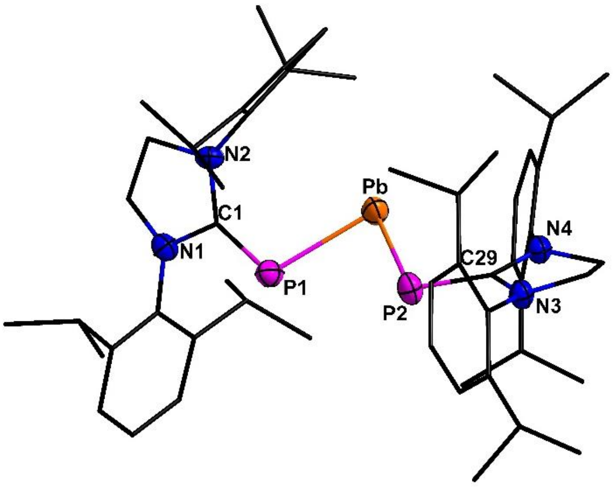

Figure S20. Molecular structure of **5**. Carbon-bound hydrogen atoms are omitted for clarity. Thermal ellipsoids for **5** represent a 50% probability level, carbon atoms are shown as sticks/wires for better visibility. Selected bond lengths /pm and angles /°: P1-Pb 258.23(26); P2-Pb 257.96(27); P1-C1 174.26(101); P2-C29 175.45(93); Pb-P1-C1 103.3(4); Pb-P2-C29 105.6(3); P1-Pb-P2 84.60(8); angle between the N1-C1-N2 and N3-C29-N4 plane 64.950(672); angle between the N1-C1-N2 and P1-Pb-P2 plane 39.602(408); angle between the N3-C29-N4 and P1-Pb-P2 plane 25.375(485).

Crystal data of 6: $C_{54}H_{76}Cl_2N_4P_2Sn_2{\cdot}3C_7H_8$, 1427.85 g·mol^{-1}, 100K, monoclinic, $P2_1/n$, a = 2536.7(3) pm, b = 1209.57(14) pm, c = 2554.3(3) pm, α = 90°, β = 114.884(3)°, γ = 90°, V = 7110.0(14) Å^3, Z = 4, ρ = 1.334 g·cm^{-3}, μ = 0.866, *F(000)* = 2968.0, *GooF* = 1.078. A total of 124517 reflections was collected of which 12521 were unique (R(int) = 0.1594). R_1 (wR_2 all data) = 0.0483 (0.0887) for 832 parameters and 9388 reflections ($I > 2\sigma(I)$). CCDC 1919177.

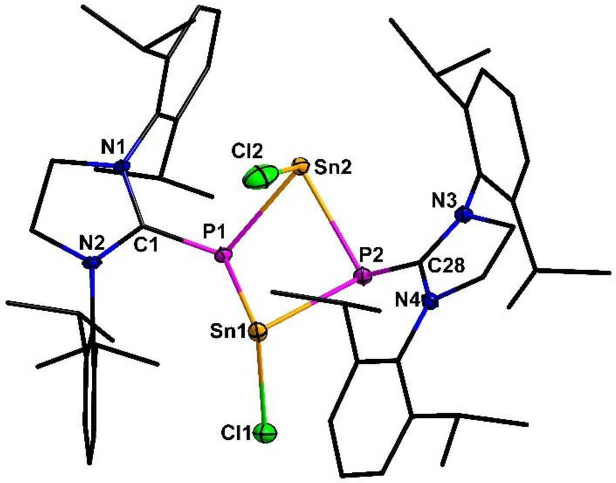

Figure S21. Molecular structure of **6**. Carbon-bound hydrogen atoms are omitted for clarity. Thermal ellipsoids for **6** represent a 50% probability level, carbon atoms are shown as balls and sticks/wires for better visibility. Selected bond lengths /pm and angles /°: P1-Sn1 264.9(10); P1-Sn2 259.55(10); P2-Sn1 266.31(13); P2-Sn2 262.90(13); Sn1-Cl1 251.48(12); Sn2-Cl2 254.75(13); P1-C1 180.11(48); P2-C28 179.88(36); C1-P1-Sn1 111.86(12); C1-P1-Sn2 107.56(12); C28-P2-Sn1 110.57(13); C28-P2-Sn2 103.69(13); P1-Sn1-P2 71.85(3); P1-Sn2-P2 73.25(3); Sn1-P1-Sn2 90.39(3); Sn1-P2-Sn2 89.38(3); P1-Sn1-Cl1 96.04(4); P2-Sn1-Cl1 96.57(4); P1-Sn2-Cl2 93.13(4); P2-Sn2-Cl2 91.77(4); angle between the P1-Sn1-P2 and the P1-Sn2-P2 plane 57.617(35); angle between the Sn1-P1-Sn2 and the Sn1-P2-Sn2 plane 66.581(40); angle between the N1-C1-N2 and the N3-C28-N4 plane 52.998(341).

Crystal data of 7: $C_{54}H_{76}Br_2N_4P_2Pb_2$, 1417.32 g·mol^{-1}, 100K, monoclinic, $P2_1$, a = 1297.65(6) pm, b = 1414.47(6) pm, c = 1554.4073) pm, α = 90°, β = 102.2910(16)°, γ = 90°, V = 2787.7(2) Å^3, Z = 2, ρ = 1.689 g·cm^{-3}, μ = 7.559, *F(000)* = 1384.0, *GooF* = 1.017. A total of 109017 reflections was collected of which 21356 were unique (R(int) = 0.0349). R_1 (wR_2 all data) = 0.0202 (0.379) for 618 parameters and 19743 reflections ($I > 2\sigma(I)$). CCDC 1919175.

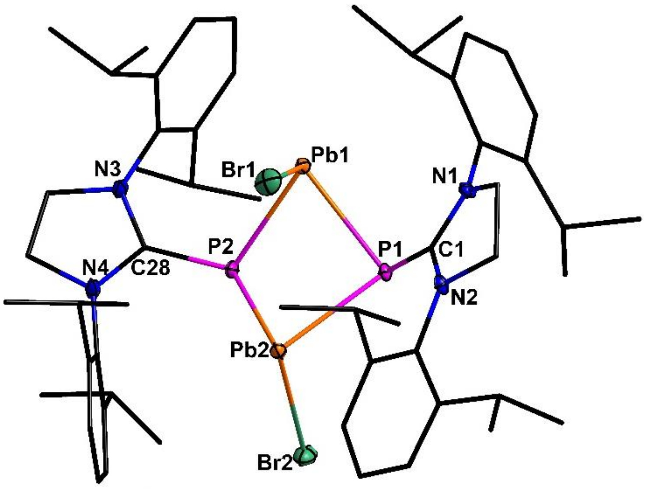

Figure S22. Molecular structure of **7**. Carbon-bound hydrogen atoms are omitted for clarity. Thermal ellipsoids for **7** represent a 50% probability level, carbon atoms are shown as balls and sticks/wires for better visibility. Selected bond lengths /pm and angles /°: P1-Pb1 269.18(8); P1-Pb2 276.82(8); P2-Pb1 268.91(8); P2-Pb2 272.48(9); Pb1-Br1 285.02(5); Pb2-Br2 280.00(5); P1-C1 179.79(28); P2-C28 179.58(29); C1-P1-Pb1 99.37(10); C1-P1-Pb2 108.57(10); C28-P2-Pb1 109.2(1); C28-P2-Pb2 119.24(10); P1-Pb1-P2 75.41(2); P1-Pb2-P2 73.60(2); Pb1-P1-Pb2 89.94(2); Pb1-P2-Pb2 90.93(2); P1-Pb1-Br1 88.898(19); P2-Pb1-Br1 97.93(2); P1-Pb2-Br2 95.634(19); P2-Pb2-Br2 92.840(19); angle between the P1-Pb1-P2 and the P1-Pb2-P2 plane 53.818(22); angle between the Pb1-P1-Pb2 and the Pb1-P2-Pb2 plane 61.510(31); angle between the N1-C1-N2 and the N3-C28-N4 plane 52.141(283).

Computational details

Analytical data at DFT level

Structures were optimized using the scalar-relativistic DLU-X2C Hamiltonian[12,13] employing the finite nucleus model as implemented in TURBOMOLE[14-16]. The BP86,[17,18] B3LYP,[19-21] PBE,[22] PBE0,[22,23] TPSS[24] and TPSSh[25] functional were selected together with fine grids (grid 4a[26]) and the x2c-TZVPall basis set[27]. For NMR studies the corresponding x2c-TZVPall-s bases[26] were chosen with the DLU-X2C Hamiltonian[28]. The multipole-accelerated resolution of the identity approximation[29,30] and the corresponding auxiliary basis sets[26] were used throughout. An SCF threshold of 10^{-9} E_h and a density threshold of 10^{-8} a.u. were selected. PH_3 serves as the reference for ^{31}P to avoid aqueous H_3PO_4. The absolute shielding constants of these two compounds are 328.35 ppm and 594.45(0.63) ppm according to Jameson and co-workers.[31] The Cartesian coordinates in atomic units of all compounds are given in a separate text file (DFT-structures.txt) as part of the ESI.

Compound 3

Table S1. Structure of compound **3**.

	BP86	PBE	TPSS	B3LYP	PBE0	TPSSh
**d(C-P) /pm**	178.03 - 178.04	177.93 - 177.95	178.00- 178.03	177.92 - 177.95	176.84 - 176.89	177.66 - 177.68
**d(P-Ge) /pm**	233.59 - 233.62	233.00 - 233.00	232.23 - 232.30	233.32 - 233.39	230.56 - 230.61	231.74 - 232.04
**∠(C-P-Ge) /°**	107.46 - 107.51	106.43 - 106.46	107.06 - 107.16	107.88 - 107.94	105.79 - 105.83	106.44 - 106.53
**∠(P-Ge-P) /°**	86.61	86.59	86.37	88.47	87.85	87.65
**^{31}P *δ* /ppm**	217	207	171	214	166	160
**^{13}C *δ* /ppm C_{NHC}**	197	198	195	210	204	199
**^{73}Ge *δ* /ppm**	1224	1171	1145	1358	1234	1190

Compound 4

Table S2. Structure of compound **4**.

	BP86	PBE	TPSS	B3LYP	PBE0	TPSSh
**d(C-P) /pm**	177.43 - 177.45	177.32 - 177.34	177.35 - 177.39	177.37 - 177.41	176.16 - 176.16	176.88 - 176.95
**d(P-Sn) /pm**	253.95 - 253.97	253.27 - 253.34	252.82 - 252.87	253.72 - 253.79	250.57 - 250.59	251.64 - 251.70
**∠(C-P-Sn) /°**	108.52 - 108.53	107.10 - 107.17	107.57 - 107.62	107.95 - 108.00	106.55 - 106.63	106.91 -107.15
**∠(P-Sn-P) /°**	85.11	85.49	85.39	88.47	86.74	86.11
**^{31}P *δ* /ppm**	220	204	166	220	156	149
**^{13}C *δ* /ppm C_{NHC}**	200	199	197	212	206	201
**^{119}Sn *δ* /ppm**	1980	1873	1816	2207	1974	1853

Compound 5

Table S3. Structure of compound **5**.

	BP86	PBE	TPSS	B3LYP	PBE0	TPSSh
**d(C-P) /pm**	176.98 - 177.03	176.84 - 176.87	176.80 - 176.84	176.89 - 177.08	175.56 - 175.57	176.30 - 176.35
**d(P-Pb) /pm**	262.70 - 262.72	262.04 - 262.08	262.00 - 262.09	262.94 - 263.35	259.19 - 259.20	260.64 - 260.89
**∠(C-P-Pb) /°**	108.90 - 109.01	107.51 - 107.67	107.76 - 107.86	107.56 - 108.01	107.30 - 107.47	107.04 - 107.28
**∠(P-Pb-P) /°**	84.96	85.15	84.98	90.03	85.91	85.91
**^{31}P *δ* /ppm**	242	222	181	250	169	161
**^{13}C *δ* /ppm C_{NHC}**	160	199	198	213	202	201
**^{207}Pb *δ* /ppm**	4287	4054	3940	4848	4238	4007

Compound 6

Table S4. Structure of compound **6**.

	BP86	PBE	TPSS	B3LYP	PBE0	TPSSh
**d(C1-P1) /pm**	178.95	178.70	179.09	178.99	177.93	178.45
**d(C28-P2) /pm**	178.92	178.93	179.06	179.21	178.62	178.81
**d(P1-Sn1) /pm**	271.90	271.02	270.95	273.89	267.34	269.85
**d(P2-Sn1) /pm**	271.90	271.21	270.98	272.45	268.02	268.64
**d(P1-Sn2) /pm**	267.44	266.18	265.82	267.40	262.89	263.77
**d(P2-Sn2) /pm**	267.32	266.86	265.76	268.88	264.85	265.42
**∠(P1-Sn1-Cl1) /°**	96.58	96.48	95.68	98.50	94.58	96.84
**∠(P2-Sn1-Cl1) /°**	96.53	96.15	95.80	95.96	94.08	94.24
**∠(P1-Sn2-Cl2) /°**	93.88	94.88	93.24	96.44	95.87	94.79
**∠(P2-Sn2-Cl2) /°**	94.21	93.12	93.51	93.26	92.46	91.96
**∠(P1-Sn1-P2) /°**	70.19	69.82	70.25	71.22	73.34	70.77
**∠(P1-Sn2-P2) /°**	71.56	71.21	71.82	72.77	74.57	72.21
**^{31}P δ /ppm**	5	7	-34	-6	-50	-45
**^{13}C δ /ppm C_{NHC}**	194	186	192	207	198	191
**^{119}Sn δ /ppm**	-18, 161	-82, 94	-114, 89	36, 193	-57, 100	-123, 59

Compound 7

Table S5. Structure of compound **7**.

	BP86	PBE	TPSS	B3LYP	PBE0	TPSSh
**d(C1-P1) /pm**	178.76	178.57	178.73	179.17	177.70	178.34
**d(C28-P2) /pm**	178.63	178.48	178.64	178.67	177.54	178.25
**d(P1-Pb1) /pm**	274.30	273.42	273.19	276.24	270.17	271.89
**d(P2-Pb1) /pm**	274.10	273.26	273.21	275.09	269.61	271.50
**d(P1-Pb2) /pm**	281.45	280.35	281.05	282.97	276.96	279.00
**d(P2-Pb2) /pm**	281.22	280.23	280.78	281.90	277.11	279.43
**∠(P1-Pb1-Br1) /°**	92.10	91.95	91.97	92.52	90.83	90.95
**∠(P2-Pb1-Br1) /°**	94.65	94.65	93.48	96.34	94.40	94.15
**∠(P1-Pb2-Br2) /°**	99.21	98.20	98.92	98.20	97.33	97.93
**∠(P2-Pb2-Br2) /°**	99.19	98.61	98.40	98.13	98.30	98.46
**∠(P1-Pb1-P2) /°**	72.84	72.82	72.38	75.84	73.32	73.22
**∠(P1-Pb2-P2) /°**	70.71	70.74	70.10	73.72	71.13	70.94
**^{31}P δ /ppm**	14	9	-24	4	-35	-40
**^{13}C δ /ppm C_{NHC}**	196	195	196	203	204	198
**^{207}Pb δ /ppm**	544, 32	461, -42	359, -202	681, 235	332, -227	332, -243

We estimated the impact of dispersive interactions by additional application of Grimme's D4 correction.[38] For the BP86 functional this leads to shortening of C-P and M-P bonds by 1-2 pm (independent of M), to reduction of ∠(C-P-M) by 3.2/5.3/6.6° for M=Ge/Sn/Pb and to – smaller - increase of ∠(P-M-P) by 0.2/1.5/4.7°. Overall, the agreement of bond angles with experimental data in the end is not better than without D4, but the (small) errors in distances are in fact almost completely corrected.

Analysis of chemical bond: Wiberg bond indices

Wiberg bond indices[32] (WBI) were calculated with the x2c-TZVPall basis set and the same methods as above. The impact of spin-orbit coupling on the WBI was studied at the TPSS level and found to be negligible as it affected the WBI typically by only 0.01.

Compound 3

Table S6. WBI of compound **3**.

	BP86	PBE	TPSS	B3LYP	PBE0	TPSSh
**WBI (C-P)**	1.39	1.38	1.29	1.43	1.39	1.33
**WBI (P-Ge)**	1.22	1.21	1.20	1.23	1.22	1.20
**HOMO-LUMO GAP /eV**	1.77	1.79	1.89	2.93	3.26	2.45

Compound 4

Table S7. WBI of compound **4**.

	BP86	PBE	TPSS	B3LYP	PBE0	TPSSh
**WBI (C-P)**	1.42	1.42	1.33	1.46	1.43	1.34
**WBI (P-Sn)**	1.15	1.14	1.13	1.15	1.14	1.13
**HOMO-LUMO GAP /eV**	1.68	1.70	1.80	2.76	3.12	2.37

Compound 5

Table S8. WBI of compound **5**.

	BP86	PBE	TPSS	B3LYP	PBE0	TPSSh
**WBI (C-P)**	1.45	1.45	1.37	1.48	1.46	1.38
**WBI (P-Pb)**	1.11	1.11	1.08	1.11	1.10	1.08
**HOMO-LUMO GAP /eV**	1.67	1.72	1.83	2.71	3.18	2.41

Compound 6

Table S9. WBI of compound **6**.

	BP86	PBE	TPSS	B3LYP	PBE0	TPSSh
**WBI (C1-P1)**	1.25	1.21	1.14	1.26	1.21	1.12
**WBI (C28-P2)**	1.25	1.21	1.14	1.25	1.20	1.14
**WBI (P1-Sn1)**	0.73	0.73	0.71	0.70	0.72	0.70
**WBI (P2-Sn1)**	0.73	0.74	0.71	0.74	0.74	0.73
**WBI (P1-Sn2)**	0.79	0.79	0.76	0.79	0.79	0.77
**WBI (P2-Sn2)**	0.79	0.78	0.76	0.77	0.76	0.75
**HOMO-LUMO GAP /eV**	2.16	2.12	2.26	3.41	3.54	2.85

Compound 7

Table S10. WBI of compound **7**.

	BP86	PBE	TPSS	B3LYP	PBE0	TPSSh
**WBI (C1-P1)**	1.26	1.24	1.15	1.27	1.21	1.15
**WBI (C28-P2)**	1.28	1.25	1.17	1.30	1.22	1.16
**WBI (P1-Pb1)**	0.78	0.78	0.75	0.76	0.78	0.75
**WBI (P2-Pb1)**	0.77	0.76	0.74	0.76	0.77	0.75
**WBI (P1-Pb2)**	0.68	0.69	0.66	0.69	0.70	0.67
**WBI (P2-Pb2)**	0.68	0.68	0.67	0.67	0.67	0.66
**HOMO-LUMO GAP /eV**	2.32	2.30	2.46	3.49	3.83	3.04

Analysis of chemical bond: MO plots of frontier orbitals

MO plots of the frontier MOs (HOMO-2 to LUMO+2) are displayed with an isovalue of 0.04 a.u. at the x2c-TZVPall/TPSS level of theory. The plots show the π character of the P-M bonds in HOMO-2 of compounds **3** to **5**. HOMO-1 corresponds to the C-P π bond. LUMO+1 and LUMO+2 display no contribution at the C-P-M system. Hydrogen atoms are omitted for clarity.

Compound 3

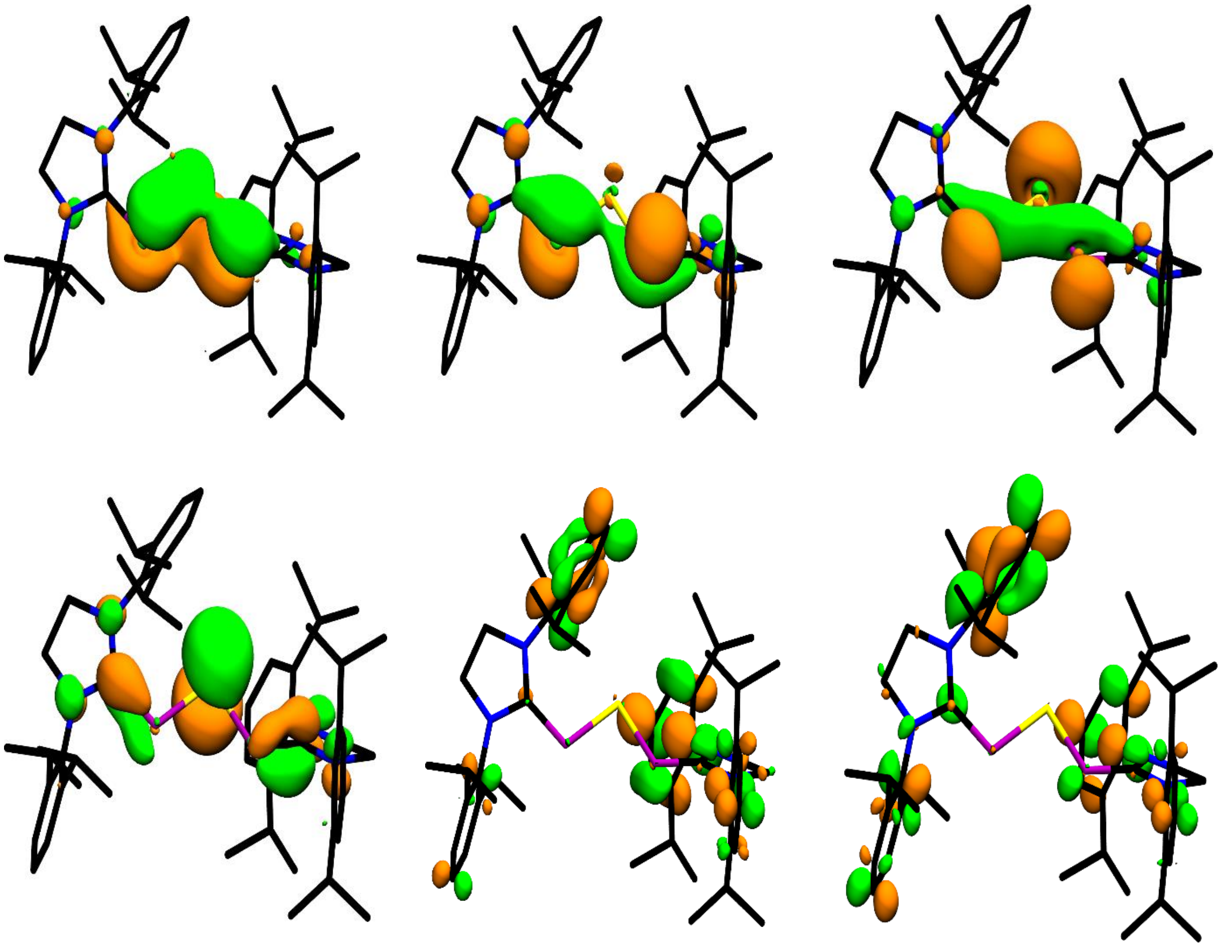

Figure S23. Frontier MOs of compound **3** with an isovalue of 0.04 a.u. (HOMO-2 at top left to LUMO+2 at bottom right).

Compound 4

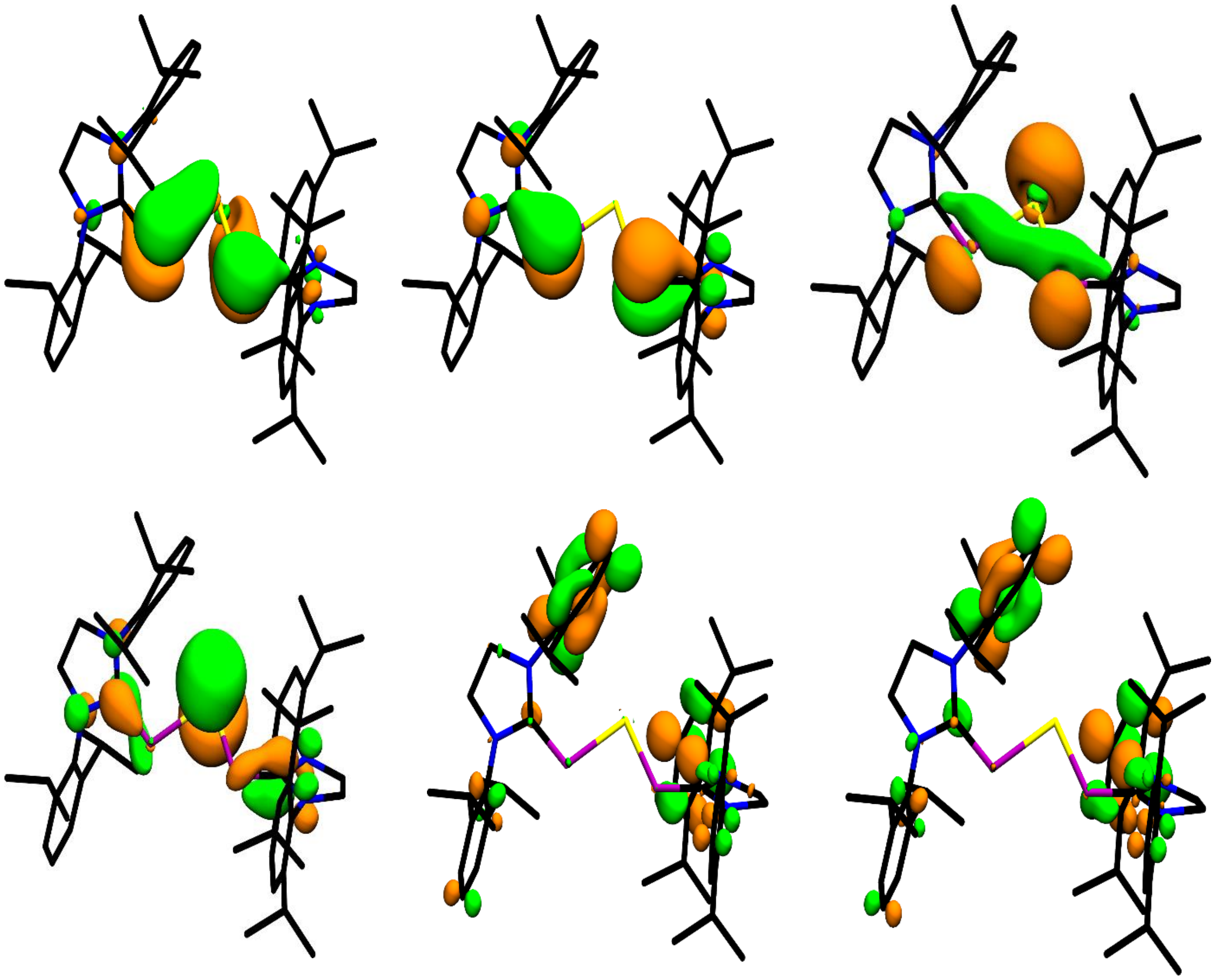

Figure S24. Frontier MOs of compound **4** (see Figure S23 for details).

Compound 5

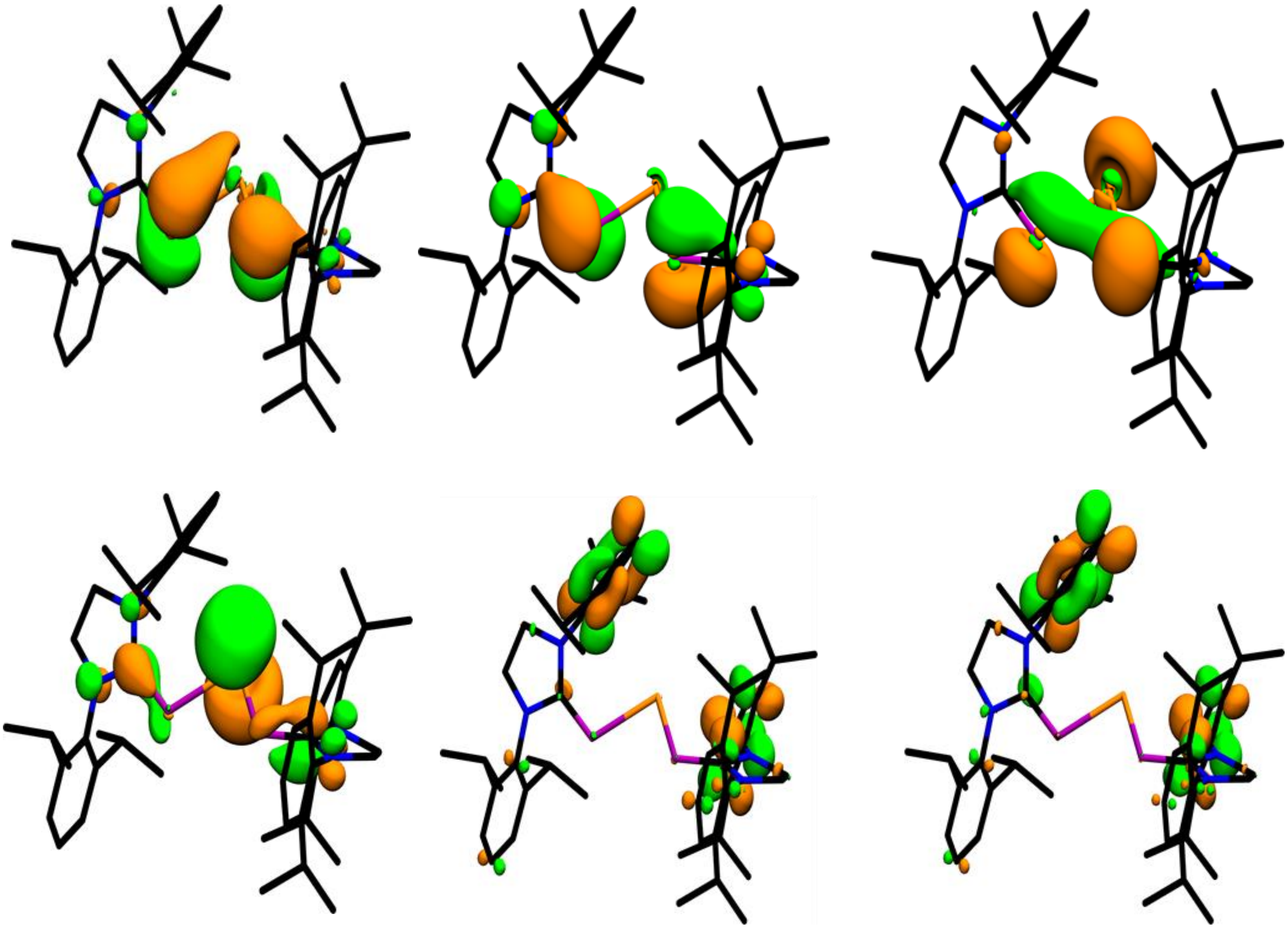

Figure S25. Frontier MOs of compound **5** (see Figure S23 for details).

Compound 6

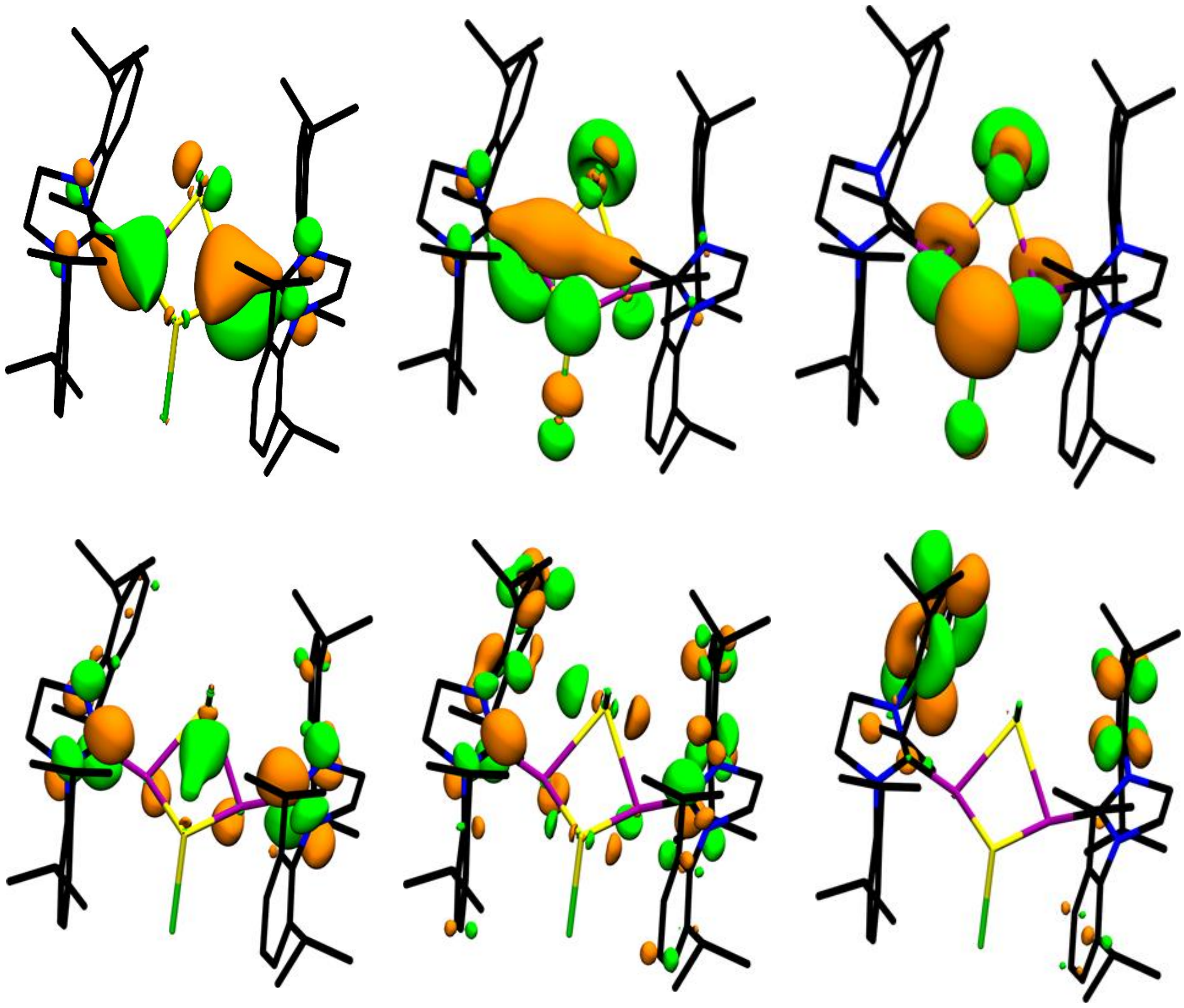

Figure S26. Frontier MOs of compound **6** (see Figure S23 for details).

Compound 7

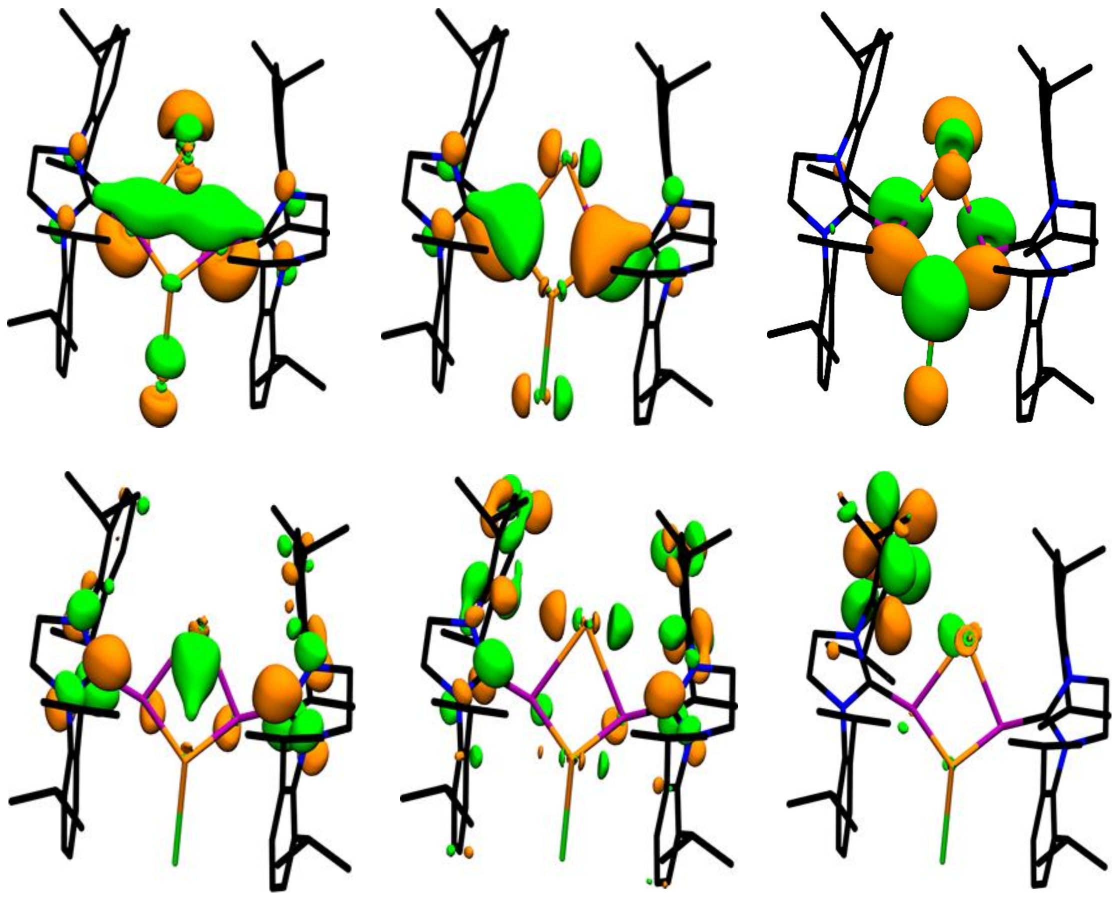

Figure S27. Frontier MOs of compound 7 (see Figure S23 for details).

Analysis of chemical bond: Natural bond orbital population

The chemical bond between the phosphorus atom and the group 14 element was further studied utilizing a natural bond orbital population analysis[33]. The TPSSh functional together with the methods stated in the last section were employed. The natural orbital population of the p orbitals of phosphorus decreases from compound **3** to **5**. The population of the p orbitals of the metal atoms shows the complementary trend. This is in line with the NMR shifts, Due to the pπ-pπ electron density is transferred from the phosphorus to the metal atom, which results in the observed NMR shifts.

Table S11. Overall population at the phosphorus atoms per orbital type.

**Compound**	s	p	d	f
**3**	5.63	9.55	0.027	0.00084
**4**	5.64	9.63	0.026	0.00080
**5**	5.65	9.64	0.023	0.00067
**6**	5.59	9.74	0.029	0.00085
**7**	5.60	9.78	0.024	0.00066

Table S12. Valence population at the phosphorus atoms per orbital type.

**Compound**	s	p	d	f
**3**	1.63	3.55	0.03	0.00
**4**	1.64	3.63	0.02	0.00
**5**	1.65	3.64	0.02	0.00
**6**	1.59	3.74	0.03	0.00
**7**	1.60	3.78	0.02	0.00

Table S13. Overall population at the tetrel atoms per orbital type.

**Compound**	s	p	d	f
**3**	7.78	13.97	10.01	0.0010
**4**	9.81	19.73	20.00	0.0019
**5**	11.88	25.57	29.99	14.00
**6**	9.82	19.45	20.00	0.0028
**7**	11.90	25.33	30.00	14.00

Table S14. Valence population at the tetrel atoms per orbital type.

**Compound**	s	P	d	f
**3**	1.78	1.97	0.01	0.00
**4**	1.81	1.73	0.01	0.00
**5**	1.88	1.57	0.00	0.00
**6**	1.81	1.45	0.01	0.00
**7**	1.90	1.32	0.00	0.00

UV-Vis spectra

The one-component TDDFT[34-36] (PBE0 and TPSSh) method was used together with the DLU-X2C Hamiltonian, the RI-J approximation and a seminumerical exchange[37] for the excited state calculations only (grid -1). The 10 lowest states in energy were considered. The position of the first absorption maximum is given below in nm. The first absorption maximum corresponds to the second excitation. The nature of the transition was studied by visualization of the transition density and the corresponding Mulliken population.

Table S15. Time-dependent density functional theory s(TDDFT) study of compound **3**-**5**. First absorption maximum is given in nm.

**Compound**	PBE0 (singlet)	PBE0 (triplet)	TPSSh (singlet)	TPSSh (triplet)
**3**	482	653	507	687
**4**	499	647	528	687
**5**	508	645	539	683

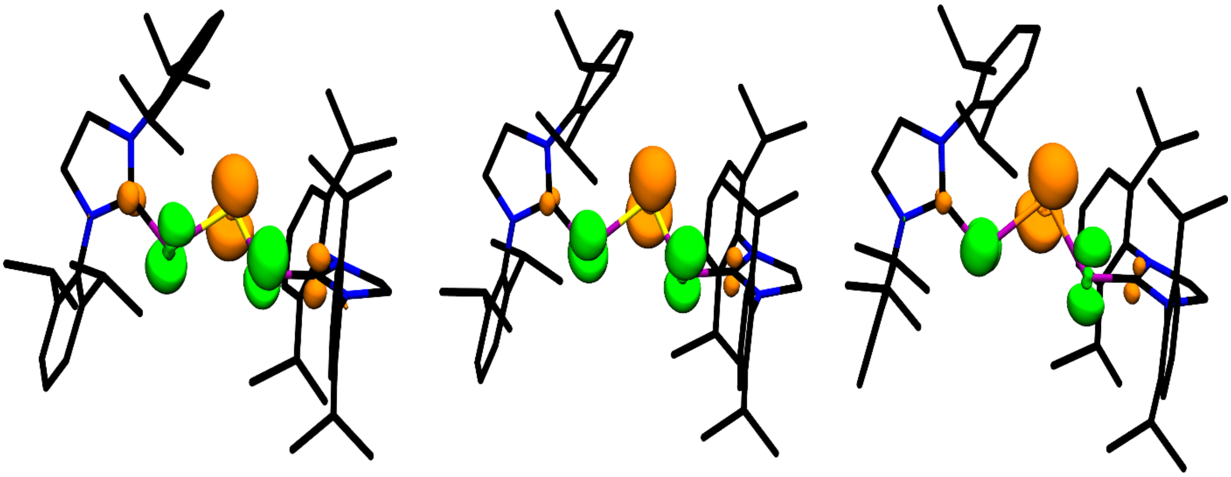

Figure S28. Transition density of compound **3** (left), **4** (middle) and **5** (right) with an isovalue of 0.005 a.u., where green denotes the initial electron density and orange the final electron density.

Comparison to ate complexes K[SIMesP$_3$M]

The WBIs and the bonding situation is compared to the recently studied K[SIMesP$_3$M] (M=Ge, Sn, Pb) complexes[3] . The structures were optimized with at the scalar-relativistic DLU-X2C/x2c-TZVPall/TPSSh level (grid 4a). The structures are given in the DFT-structures.txt file.

Table S16. WBI of the P-M bonds of K[SIMesP$_3$M] (M = Ge, Sn, Pb). The indices of the individual P-M bonds were averaged.

**M**	WBI TPSSh
**Ge**	0.85
**Sn**	0.83
**Pb**	0.78

References

[1] K. M. Kuhn and R. H. Grubbs, *Org. Lett.*, 2008, **10**, 2075–2077.

[2] F. F. Puschmann, D. Stein, D. Heift, C. Hendriksen, Z. A. Gal, H.-F. Grützmacher and H. Grützmacher, *Angew. Chem. Int. Ed.*, 2011, **50**, 8420–8423.

[3] F. F. Puschmann, D. Stein, D. Heift, C. Hendriksen, Z. A. Gal, H.-F. Grützmacher and H. Grützmacher, *Angew. Chem.*, 2011, **123**, 8570–8574.

[4] P. J. Bailey, R. A. Coxall, C. M. Dick, S. Fabre, L. C. Henderson, C. Herber, S. T. Liddle, D. Loroño-González, A. Parkin and S. Parsons, *Chem. Eur. J.*, 2003, **9**, 4820–4828.

[5] M. Balmer, F. Weigend and C. von Hänisch, *Chem. Eur. J.*, 2019, **25**, 4914–4919.

[6] M. Cicač-Hudi, J. Bender, S. H. Schlindwein, M. Bispinghoff, M. Nieger, H. Grützmacher and D. Gudat, *Eur. J. Inorg. Chem.*, 2016, **2016**, 649–658.

[7] M. Bispinghoff, A. M. Tondreau, H. Grützmacher, C. a. Faradji and P. G. Pringle, *Dalton Trans.*, 2016, **45**, 5999–6003.

[8] O. Lemp, M. Balmer, K. Reiter, F. Weigend and C. von Hänisch, *Chem. Commun.*, 2017, **53**, 7620–7623.

[9] G. M. Sheldrick, *Acta Crystallogr. Sect. A Found. Adv.*, 2015, **71**, 3–8.

[10] G. M. Sheldrick, *Acta Crystallogr. Sect. C Struct. Chem.*, 2015, **71**, 3–8.

[11] O. V. Dolomanov, L. J. Bourhis, R. J. Gildea, J. A. K. Howard and H. Puschmann, *J. Appl. Crystallogr.*, 2009, **42**, 339–341.

[12] D. Peng, N. Middendorf, F. Weigend and M. Reiher, *J. Chem. Phys.*, 2013, **138**, 184105.

[13] Y. J. Franzke, N. Middendorf and F. Weigend, *J. Chem. Phys.*, 2018, **148**, 104110.

[14] R. Ahlrichs, M. Bär, M. Häser, H. Horn, and C. Kölmel, *Chem. Phys. Lett.*, 1989, **162**, 165–169.

[15] F. Furche, R. Ahlrichs, C. Hättig, W. Klopper, M. Sierka, and F. Weigend, *Wiley Interdiscip. Rev.: Comput. Mol. Sc*i., 2014, **4**, 91–100.

[16] TURBOMOLE V7.4 2019, a development of University of Karlsruhe and Forschungszentrum Karlsruhe GmbH, 1989-2007, TURBOMOLE GmbH, since 2007; available from http://www.turbomole.com.

[17] A. D. Becke, *Phys. Rev. A: At., Mol., Opt. Phys*., 1988, **38** 1988, 3098–3100.

[18] J. P. Perdew, *Phys. Rev. B: Condens. Matter Mater. Phys*., 1986, **33**, 8822–8824.

[19] A. D. Becke, *J. Chem. Phys*., 1993, **98**, 5648–5652.

[20] C. Lee, W. Yang and R. G. Parr, *Phys. Rev. B: Condens. Matter Mater. Phys.*, 1988, **37**, 785–789.

[21] P. J. Stephens, F. J. Devlin, C. F. Chablowski and M. J. Frisch, *J. Phys. Chem*., 1994, **98**, 11623–11627.

[22] J. P. Perdew, K. Burke and M. Ernzerhof, *Phys. Rev. Lett*., 1996, **77**, 3865–868.

[23] C. Adamo and V. Barone, *J. Chem. Phys.*, 1999, **110**, 6158–6170.

[24] J. Tao, J. P. Perdew, V. N. Staroverov and G. E. Scuseria, *Phys. Rev. Lett,*. 2003, **91**, 146401.

[25] V. N. Staroverov, G. E. Scuseria, J. Tao and J. P. Perdew, *J. Chem. Phys*., 2003, **119**, 12129–12137.

[26] Y. J. Franzke, R. Treß, T. M. Pazdera and F. Weigend, *Phys. Chem. Chem. Phys*., 2019, **21**, 16658–16664.

[27] P. Pollak and F. Weigend, *J. Chem. Theory Comput*., 2018, **13**, 3696–3705.

[28] Y. J. Franzke and F. Weigend, *J. Chem. Theory Comput*., 2019, **15**, 1028–1043.

[29] M. Sierka, A. Hogekamp and R. Ahlrichs, *J. Chem. Phys*., 2003, **118**, 9136–9148.

[30] K. Reiter, F. Mack and F. Weigend, *J. Chem. Theory Comput*., 2018, **14**, 191–197.

[31] C. J. Jameson, A. De Dios and A. K. Jameson, *Chem. Phys. Lett*., 1990, **167**, 575–582.

[32] K. B. Wiberg, *Tetrahedron*, 1968, **24**, 1083–1096.

[33] A. E. Reed, R. B. Weinstock and F. Weinhold, *J. Chem. Phys*., 1985, **83**, ,83(2), 735–746.

[34] R. Bauernschmitt and R. Ahlrichs, *Chem. Phys. Lett.*, 1996, **256**, 454–464.

[35] R. Bauernschmitt, M. Häser, O. Treutler and R. Ahlrichs, *Chem. Phys. Lett.*, 1997, **264**, 573–578.

[36] S. Grimme, F. Furche and R. Ahlrichs, *Chem. Phys. Lett.*, 2002, **361**, 321–328.

[37] P. Plessow and F. Weigend, *J. Comput. Chem*., 2012, **33**, 810–816.

[38] E. Caldeweyher, C. Bannwarth and S. Grimme, *J. Chem. Phys.*, 2017, **147**, 34112.
